# Calcite Surfaces Modified with Carboxylic Acids (C_2_ to C_18_): Layer Organization, Wettability, Stability, and Molecular Structural Properties

**DOI:** 10.1021/acs.langmuir.3c01252

**Published:** 2023-10-12

**Authors:** Natalia A. Wojas, Eric Tyrode, Robert Corkery, Marie Ernstsson, Viveca Wallqvist, Mikael Järn, Agne Swerin, Joachim Schoelkopf, Patrick A. C. Gane, Per M. Claesson

**Affiliations:** † RISE Research Institutes of Sweden, Division of Bioeconomy and Health–Material and Surface Design, Box 5607, SE-114 86 Stockholm, Sweden; ‡ KTH Royal Institute of Technology, Department of Chemistry, Teknikringen 30 SE, 11428 Stockholm, Sweden; § Australian National University Department of Applied Mathematics, Research School of Physics and Engineering, Canberra ACT 0200, Australia; ∥ Karlstad University Faculty of Health Science and Technology, Department of Engineering and Chemical Sciences: Chemical Engineering, SE-651 88 Karlstad, Sweden; ⊥ Omya International AG, Baslerstrasse 42, CH-4665 Oftringen, Switzerland; # Aalto University School of Chemical Engineering, Department of Bioproducts and Biosystems, P.O. Box 16300, FI-00076 Aalto, Finland; ¶ University of Belgrade, Faculty of Technology and Metallurgy, Karnegijeva 4, 11200 Belgrade, Serbia

## Abstract

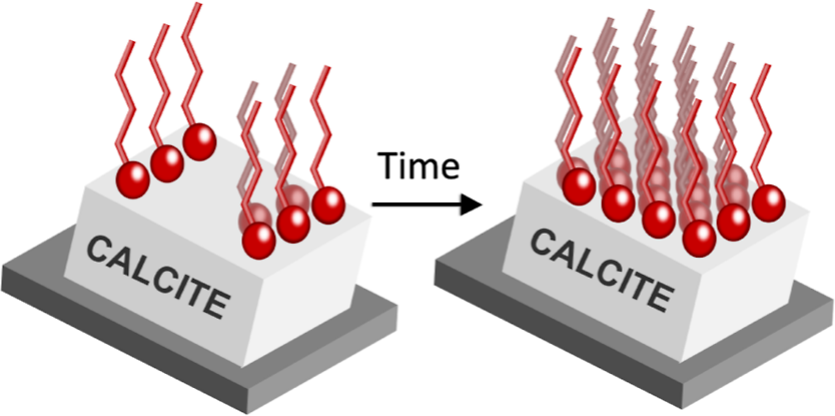

A fundamental understanding of the interactions between mineral surfaces and amphiphilic surface modification agents is needed for better control over the production and uses of mineral fillers. Here, we controlled the carboxylic acid layer formation conditions on calcite surfaces with high precision via vapor deposition. The properties of the resulting carboxylic acid layers were analyzed using surface-sensitive techniques, such as atomic force microscopy (AFM), contact angle measurements, angle resolved X-ray photoelectron spectroscopy (XPS), and vibrational sum-frequency spectroscopy. A low wettability was achieved with long hydrocarbon chain carboxylic acids such as stearic acid. The stearic acid layer formed by vapor deposition is initially patchy, but with increasing vapor exposure time, the patches grow and condense into a homogeneous layer with a thickness close to that expected for a monolayer as evaluated by AFM and XPS. The build-up process of the layer occurs more rapidly at higher temperatures due to the higher vapor pressure. The stability of the deposited fatty acid layer in the presence of a water droplet increases with the chain length and packing density in the adsorbed layer. Vibrational sum frequency spectroscopy data demonstrate that the stearic acid monolayers on calcite have their alkyl chains in an all-trans conformation and are anisotropically distributed on the plane of the surface, forming epitaxial monolayers. Vibrational spectra also show that the stearic acid molecules interact with the calcite surface through the carboxylic acid headgroup in both its protonated and deprotonated forms. The results presented provide new molecular insights into the properties of adsorbed carboxylic acid layers on calcite.

## Introduction

Amorphous and semicrystalline polymer materials are commercially used in many applications, such as in construction, plastics, paints, adhesives, sealants, and agricultural industries.^1^ However, to enhance their functional properties (e.g., enlarged volume, rheological modification, mechanical reinforcement, or optical characteristics^1,2^) without negative impacts on the cost, they are often mixed with various particulate additives, including crystalline minerals, so-called mineral fillers.^3,4^ Thus, adding such mineral fillers (mostly nano- and microsized particles with a high surface area^5^) leads to new desirable properties of the final product.^3^ Among mineral fillers, calcium carbonate (CaCO_3_) is known as one of the most functionally cost-effective.^6^ Furthermore, it is nontoxic, odorless, has a high chemical purity, and conforms, in such a use, to food contact legislation.^1^ It is regarded as the oldest manufactured powder, and used in numerous industries.^3^ This most abundant simple salt on Earth can be obtained in form of CaCO_3_ powder by grinding sedimentary rocks (mostly chalk, limestone, and marble), and the product is termed ground calcium carbonate (GCC).^1^ Alternatively, precipitated calcium carbonate (PCC) can be prepared by carbonation of a calcium hydroxide (Ca(OH)_2_) solution.^7^ However, regardless of how it is produced, the pure surface of calcium carbonate is dynamic and its chemical state is readily affected by the environment.^8,9^ CaCO_3_ is naturally wetted by water, either as liquid or as humidity in the air, and exposure to water leads to dissolution and reprecipitation.^8^

Water molecules interact strongly with the CaCO_3_ surface by electrostatic interactions between calcium atoms at the surface and oxygen atoms from water and by hydrogen bonding with the protruding O atoms of the surface carbonate groups.^10,11^ The concentration of calcium ions and dissolved CO_2_ determines the surface charge in aqueous solution, and it has been reported that under common conditions, the surface is positively charged over a broad pH interval.^12−14^ This facilitates the electrostatically driven adsorption of dissociated acidic components and anionic surfactants. As a result, clean hydrophilic calcium carbonate surfaces become more hydrophobic as molecules of organic material adsorb.^8,10^ Physicochemical transformations occurring at the surface are important to countless industrial processes; therefore, solutions for preventing undesired interactions have been extensively investigated.^1−3,15−17^

The foremost challenges of using CaCO_3_ fillers are not only to overcome the dynamic nature of the CaCO_3_ surface that could result in uncontrolled, inhomogeneous aggregation or dissolution,^5,18^ but also to achieve a uniform spatial distribution and dimensional stability of CaCO_3_ particles in a polymer matrix.^5,15^ The high surface energy and hydrophilic surface of CaCO_3_ are incompatible with a low-energy surface of a nonpolar polymer matrix, where an effective way for improving compatibility is to reduce the surface energy of CaCO_3_ by surface modification.^4,5^ Such modifications diminish interactions between the filler particles dispersed in the matrix and hence improve filler dispersion and adhesion to the polymer matrix,^17,19^ while retaining processing properties at high filler content, such as optimal strain energy release, impact strength, and stiffness.^1,20,21^ In order to achieve full benefits from modified mineral fillers, especially for moisture curing applications, it is essential to minimize the pickup of undesired molecules. Particular attention needs to be given to water, as a water film alters the adsorption of modifiers^22^ and initiates undesired generation of hydrated calcium bicarbonate.^15^ Therefore, a clean, preferably freshly fractured CaCO_3_ surface with accessible positively charged calcium sites should be used for effective surface hydrophobization.^2^

In light of the above, the modification should preferably be carried out immediately after surface fracture using surface-active organic modifiers, e.g. glycols, alcohols, phosphonates, and sulfonates.^6^ Among these organic modifiers, the long-chain carboxylic acids, so-called fatty acids, are the most prominent ones in use.^2,23^ In contrast to the nonadsorbates and weak adsorbates that have slight or no effect on the CaCO_3_ dissolution rates, the fatty acids adsorb strongly to the carbonate surface, isolating it from the environment thereby reducing or even inhibiting the dissolution.^24^ However, recent atomic force microscopy (AFM) studies have shown that water droplets may cause significant changes in the adsorbed layer below the droplet and at the droplet edge.^25^ They can form thermodynamically very stable and resistant surface layers with closely packed long linear hydrophobic “tails” that presumably arrange perpendicularly to the surface with very well connected hydrophilic “head groups” with an area of 20.5 Å^2^, matching closely the available calcium sites with an area of 20.8 Å^2^.^2,3,26^ Therefore, only one molecule of fatty acid is expected to attach to each calcium ion.^4,16^ When full coverage has been reached, the adsorbed layer also has the ability to reduce abrasive wear of the underlying calcite surface.^27^ Adsorption of long-chain fatty acids on carbonate rocks is favored by their low solubility in water and their high affinity to the CaCO_3_ surface.^26^ The properties of the fatty acid layer depend strongly on pH, where the layer is adsorbed effectively as carboxylate anions only at pH values above 4.5–5.

There are primarily two methods utilized in the industry to modify CaCO_3_ surfaces: either a “dry” or a “wet/solution” treatment.^5,19^ The dry method involves high shear mixing of CaCO_3_ particles with surface modifiers at a temperature matching or exceeding the modifier’s melting point.^5,6,19^ It is proposed that the dry surface modification is driven by chemisorption of carboxyl groups onto primarily surface centers of calcium ions,^2,19^ where the dissociated hydrogen ions attach to the carbonate sites.^23^ Thus, calcium carboxylate and bicarbonate are formed.^23^ The desired monolayer with hydrocarbon chains, in principle, close to vertically oriented to the calcite surface, is achieved at a specific modifier concentration that depends on the treatment method chosen.^3,4,15,16,23^

The wet method involves a hot aqueous slurry of a mineral filler to which a hot concentrated suspension of organic modifier is added.^5,19^ Here, the solvent medium is either water or alcohol.^6^ It is recognized that an electrical double layer is formed at the calcite–water interface, and the nature of this depends on the concentration of Ca^2+^ and CO_3_^2–^ potential-determining ions, pH, and the presence of other ions. It has been suggested that H^+^ and OH^–^ ions are chemisorbed from the electrical double layer creating Ca(OH)^+^ and HCO_3_^–^ surface groups, while fatty carboxylate ions (e.g., stearate) are either chemisorbed on Ca^2+^ ions or participate in OH^–^ ion exchange,^23^ leading to the formation of calcium stearate bicarbonate^4,16^
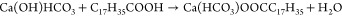

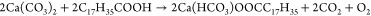


The packing efficiency, layer organization, and stability of the carboxylic acid layer are important for the efficacy of the treatment. However, it remains a largely unresolved issue, a fact that inspired this study. We utilized a vapor deposition method that allows control of the layer formation conditions with high precision. To this end, we prepared and characterized CaCO_3_ surfaces modified by carboxylic acids with chain lengths ranging from C_2_ to C_18_ under a controlled environment, varying exposure times and temperatures. It has been reported that vapor deposition on other crystalline materials, such as NaCl, generates relatively heterogeneous coatings with monolayer islands.^28^ Thus, we aimed to gain a deeper understanding of the modified surface interaction, including layer build-up, desorption, and rearrangement of the adsorbed carboxylic acids under air exposure or in contact with water droplets. To determine the structure of the fatty acid coating on CaCO_3_ surfaces, highly surface sensitive techniques, such as AFM, contact angle measurements (CA), X-ray photoelectron spectroscopy (XPS), and vibrational sum-frequency spectroscopy (VSFS), were utilized.

## Experimental Section

### Materials

In order to obtain similar surfaces in all experiments, cleaved calcite crystals were used rather than the calcium carbonate powder. To this end, optical quality calcite (Geocity AB, Stockholm, mined in Madagascar) was cleaved with a stainless-steel chisel and hammer^8^ along the dominant (101̅4) cleavage plane that generates a polar hydrophilic surface.^10,29^ The sizes of the samples were approximately 50–100 mm^2^ for atomic force microscopy, 25–400 mm^2^ for contact angle measurements, about 100 mm^2^ for X-ray photoelectron spectroscopy studies, and 100–200 mm^2^ for the vibrational sum frequency spectroscopy studies. The samples were, in all cases, approximately 3 mm thick. Areas with no evidence of excessive microcracks and steps were chosen for the measurements. Immediately after cleavage, the surfaces were purged with pressurized nitrogen gas (nitrogen > 99.9 vol %, oxygen < 20 ppm, and water < 10 ppm) to remove debris from the fractured surface and to minimize adsorption of airborne molecules. Ultrapure Milli-Q water (type 1, ASTM D1193-91) was used for wettability studies. Epoxy glue (Bostik) was used for sample attachment to the magnetic disc used in AFM studies and double adhesive conductive tape for sample attachment to the XPS holder, while for VSFS, samples were placed on a cleaned Teflon platform connected to a rotational stage.

The following saturated carboxylic acids were used for investigation of adsorption to the cleaved calcite surface: ethanoic acid (C_2_, acetic acid), ACS reagent ≥99.7% (Sigma-Aldrich); butanoic acid (C_4,_ butyric acid), ≥99%, food-grade (Sigma-Aldrich); octanoic acid (C_8_, caprylic acid), for synthesis ≥99.0% (Sigma-Aldrich); dodecanoic acid (C_12_, lauric acid): for synthesis ≥99.0%, food-grade (Sigma-Aldrich); and octadecanoic acid (C_18_, stearic acid), for synthesis ≥97.0% (Sigma-Aldrich). Silica gel granulate (Merck) was used in order to keep low humidity conditions (below 10% relative humidity) during storage and carboxylic acid vapor exposure at room temperature. The relative humidity (RH) was measured with an external sensor (HMT317, Vaisala) placed in the proximity of the calcite samples.

### Methods

#### Vapor Deposition of Carboxylic Acid

The calcite surfaces were modified by exposure to the vapor of the chosen carboxylic acid. The kinetics of the layer formation process increases with increasing vapor pressure. The vapor pressures for the different carboxylic acids at the temperatures used were calculated from measured vaporization enthalpies (see Section 1 in the Supporting Information).

##### Calcite Surface Modification by C_2_, C_4_, and C_8_ Carboxylic acids

Approximately, 45 mL of liquid carboxylic acid was poured into a flat 80 mL glass beaker with a diameter of 57 mm to ensure a large evaporation area. The beaker was then placed on a porcelain plate inside a glass desiccator (inside volume of 2.3 ± 0.2 L) at room temperature, while a beaker with silica gel granules was placed underneath. The setup was sealed and left to equilibrate for ∼1 h to stabilize the water and carboxylic acid vapor pressures before placing the calcite samples next to the beaker containing the carboxylic acid. The samples were then left inside the closed desiccator for 4 h.

##### Calcite Surface Modification by C_12_ and C_18_ Carboxylic acids

Because these carboxylic acids are solids and have low vapor pressures at room temperature, they need to be heated to achieve a sufficiently high vapor pressure. About 20 to 25 g of the corresponding carboxylic acid were first placed in a similar glass beaker as used for the shorter chain carboxylic acids. The beaker was then placed inside a 1.5 L glass box with a cover and put inside an oven (UF 55, Memmert) at a temperature above the melting point of the carboxylic acid (the melting point is 43.8 °C for the C_12_ carboxylic acid, and 69.3 °C for the C_18_ carboxylic acid, as shown in Table S3 in the Supporting Information). Silica^30^ gel was not necessary because the higher temperature allowed maintaining humidity levels below 2.5% RH. The beaker was kept in the oven for about 90 min in order to reach the desired temperature and stabilize the vapor pressure of the melted acid granules. Afterward, the oven was briefly opened to insert the calcite samples. The C_12_ samples were left inside for 4 h, while those for C_18_ were left for periods of 10, 20, or 30 min and 1, 4, or 24 h.

### Experimental Techniques

#### Contact angles

The interactions between modified calcite surfaces and water were measured using an optical contact angle device (OCA40, Data Physics Instruments GmbH) equipped with a standard operating table and automated micropipette with a disposable silicone oil- and latex-free syringe of volume 1.0 mL (Injekt-F Solo, B. Braun Melsungen AG) and a passivated stainless-steel tip of inner diameter 0.15 mm (30 GA GP, Optimum, Nordson EFD). The droplet shape was recorded by a high resolution CCD camera at a rate of 6 frames/s. The average of the CAs on the left and right sides of the droplet was calculated using the ellipse fitting method in the SCA20 software (DataPhysics Instruments GmbH).

All CA measurements were carried out just after the surface treatment process was completed. At least five calcite samples were analyzed in parallel for each condition, where each sample could fit 2 to 4 independent water droplets. After the deposition of a 1 μL droplet, the contact angle was measured as a function of time. The initial value was set at 1.2 s from the droplet contact with the surface, while for stearic acid, a semistatic contact angle was read after 30 s, as the initial fast spreading of the droplet at this stage was complete. All measurements were carried out under ambient air, where the temperature varied between 21.5 and 24.5 °C, and the humidity was around 50% RH.

#### AFM Analysis

The MultiMode 8 AFM (Bruker) with a standard holder and scanner (S/N: 10578JVLR, Bruker) was used for the evaluation of morphology and nanomechanical properties of carboxylic acid-modified calcite surfaces. For these studies, the peak force quantitative nanoscale mechanical characterization (QNM) mode was used with the selected applied force of 20 nN. Such a high force was needed in order to deform the calcite surface sufficiently for nanomechanical measurements. It was applicable without tip damage (as judged by imaging rough surfaces before and after measurements) by using an AFM probe with “Hard Diamond-Like-Carbon” tip coating (HQ/NSC35/Hard/Al BS, Mikromasch) with a nominal tip diameter of ≤20 nm. The normal spring constant, *k*_*z*_, was calculated using the thermal tune method^31^ and found to be in the range of 16.7–17.2 N/m, while from the Sader calibration method,^31^ the spring constant was found to be 17.3–17.4 N/m. We conclude that both methods give similar results. With a sapphire calibration sample (Bruker), the deflection sensitivity measured in air was found to be in the range of 22.4 to 24.1 nm/V. The indentation depth stayed within the range of 0.5–1 nm during nanomechanical measurements. The effective tip radius of the AFM probe was determined to be 6.0–7.7 nm (at 0.5 nm indentation depth) using a titanium polycrystalline-calibrated roughness sample (RS-12M, Bruker).

The modified calcite surfaces were studied directly after preparation. All measurements were carried out under ambient air, i.e. as for the CA studies. The sample scan size was set to 1 or 5 μm, with a scanning frequency of 1.0 Hz (Peak-Force frequency of 2.0 kHz), and the number of pixels in the image was 512 × 512.

Adhesion and deformation images were extracted from retraction force curves using the NanoScope Analysis program (Bruker). Deformation can be measured quantitatively and from this and the compressed layer thickness, an undisturbed layer thickness can be estimated using the “Image Math” function in the analysis software. In all cases, no image enhancement was performed, apart from flattening of height (1st order) images. The ImageJ software was used to calculate the surface coverage by domains in different environments. In addition, color level thresholding on adhesion scans was performed.

#### XPS Analysis

XPS was used to provide quantitative information on the chemical composition of the outermost 2–10 nm of the calcite surface modified with stearic acid, which is the most relevant carboxylic acid from the industrial application point of view. The instrument used was a Kratos AXIS Ultra^DLD^ X-ray photoelectron spectrometer (Kratos Analytical) operating in ultrahigh vacuum during surface analysis (pressure below 1.33 × 10^–5^ Pa). The samples were analyzed using a monochromatic Al Kα X-ray radiation source (*h*ν = 1486.6 eV) operated at 15 kV/10 mA. The analyzed sample area was below 1 mm^2^ (with most of the signal coming from an area of 700 × 300 μm^2^). Charge compensation of insulating calcite samples was achieved using the instrument neutralizer system. The takeoff angle for the photoelectrons was in most cases 90° from the surface, except in the angle-resolved measurements where lower angles were utilized to enhance the surface sensitivity further.

Leaving the samples under ultrahigh vacuum overnight without X-ray irradiation did not significantly change spectra (see Section 3 in Supporting Information and Figure S6). Nevertheless, all of the samples were analyzed directly after their preparation. The different elements present in the surface region were detected from wide scans (160 eV pass energy, 1 eV step size, and 1 sweep taking 180 s). Detailed spectra of elements of interest were collected to obtain the relative surface compositions (80 eV pass energy, 0.1 eV step size, 1 sweep taking 60–80 s), and these spectra were used for further calculations. The primary peaks analyzed were as follows: C 1s, Ca 2p, and O 1s, with relative sensitivity factors of 0.278, 1.833, and 0.780, respectively (as supplied by Kratos Analytical). High-resolution C 1s spectra (20 eV pass energy, 0.1 eV step size, and 1 sweep taking about 70 s) were recorded to determine the chemical shifts in the carbon C 1s signals due to the influence of different functional groups. Short sweep times were used to minimize the X-ray irradiation time (below 10 min for full sample analysis), and the spectra used for quantification were recorded first. The possible breakdown and/or desorption of stearic acid due to X-ray irradiation and sample heating was investigated by recording detailed spectra of Ca 2p and C 1s sequentially and repeatedly for approximately 90 min. The data were analyzed by plotting the C 1s/Ca 2p atomic ratio vs irradiation time. These results are presented in Section 3 of the Supporting Information.

The XPS data were evaluated with a Kratos Vision Processing program, and the details concerning adsorbed layer thickness and adsorbed amount calculations are presented in Section 3 of the Supporting Information.

#### VSFS Analysis

VSFS is an intrinsic surface-specific technique that provides structural and orientational information on interfacial molecules with a submonolayer sensitivity.^32^ A VSF spectrometer used in these studies has been described in detail elsewhere.^33^ Briefly, a Ti/sapphire amplifier (∼90 fs, 1 kHz, ∼ 6 W, Integra-C, Amplitude Technologies, France) is used to pump a HE-Topas (Light Conversion, Lithuania) to obtain a broadband tunable IR pulse, and a bandwidth tunable 805 nm ps pulse. In these experiments, the visible and IR beams were focused on the sample position in a copropagating geometry at angles of incidence of 70 and 55°, with average powers of ∼15 and ∼3 mW, and beam sizes (minor axis of the ellipse) at the sample position of ∼300, and ∼150 μm, respectively. The resolution was set to <3 cm^–1^, and spectra were recorded in the SSP (SF-visible-IR), PPP, SPS, SSS, and SPP polarization combination and normalized by the nonresonant PPP SF response from a gold surface.^33^ The spectrometer features a modified white light microscope for sample positioning, which allows alignment of the cleaved calcite crystal with micrometer precision and selecting areas with minimum steps or microcracks capable of generating a specular reflection of the incident visible beam. For performing, the azimuthal rotation of the (101̅4) plane, the calcite samples were located on a graduated rotational stage with an accuracy better than ±0.5°. Fine adjustment of the sample tilt was required upon rotation, as the underlying calcite samples were not totally flat. However, the area irradiated during the azimuthal rotation was ensured to be the same with a lateral precision better than 10 μm, as determined from the white light microscope images. The azimuthal angles reported are relative to the [010] crystallographic direction (see sketch in inset of Figure 7a).

**Figure 1:**
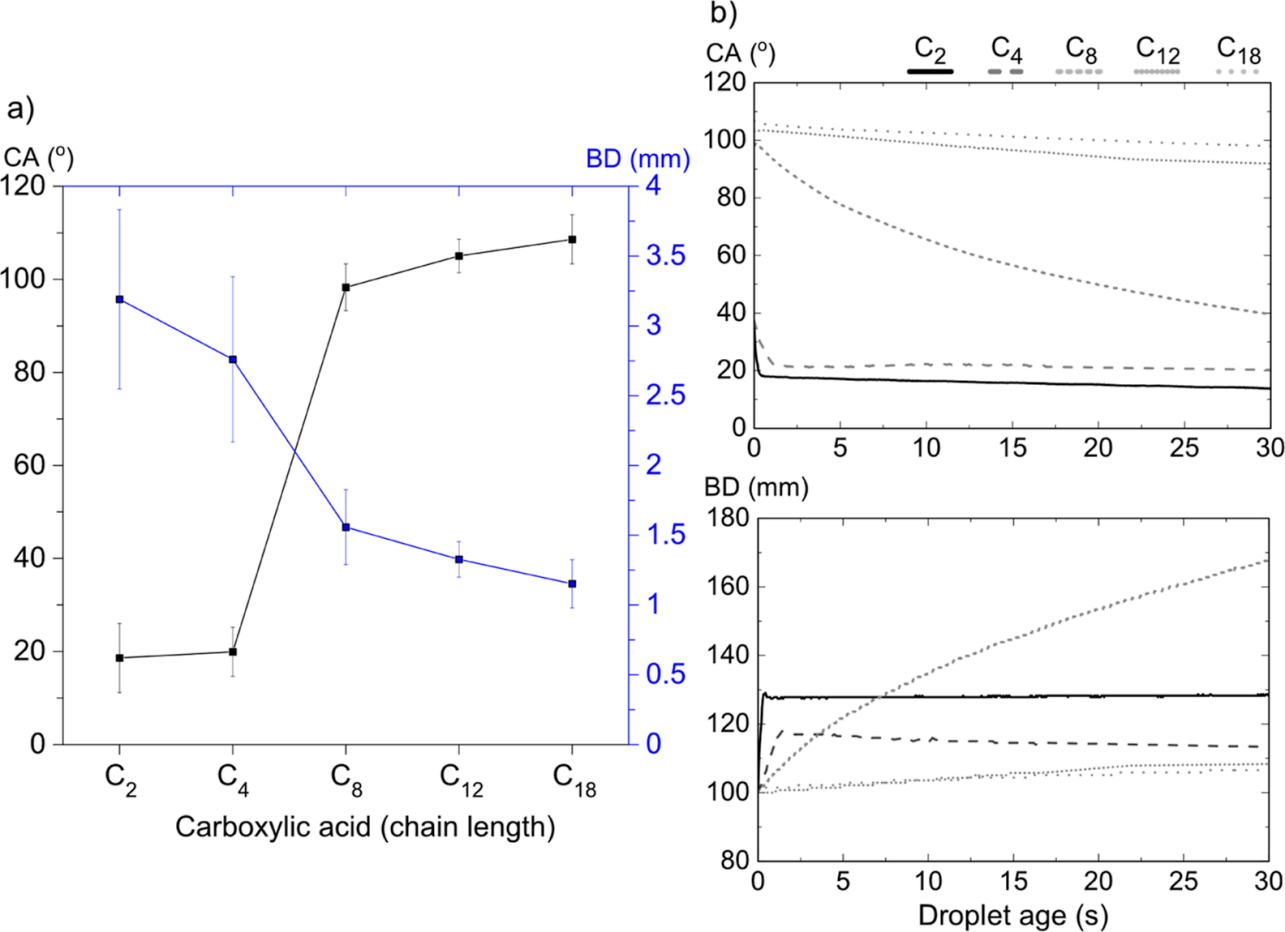
(a) Initial contact angle (CA) and base diameter (BD) of a 1 μL water droplet on calcite exposed to the vapor of increasing chain length carboxylic acids for 4 h. The modification was carried out at room temperature for C_2_, C_4_, and C_8_, at 80 °C for C_12_ and at 105 °C for C_18_. Data were evaluated 1.2 s after dispensing the droplet. The error bars represent the standard deviation of at least five different measurements. (b) CA and normalized base diameter for the same carboxylic acids as functions of time.

**Figure 2:**
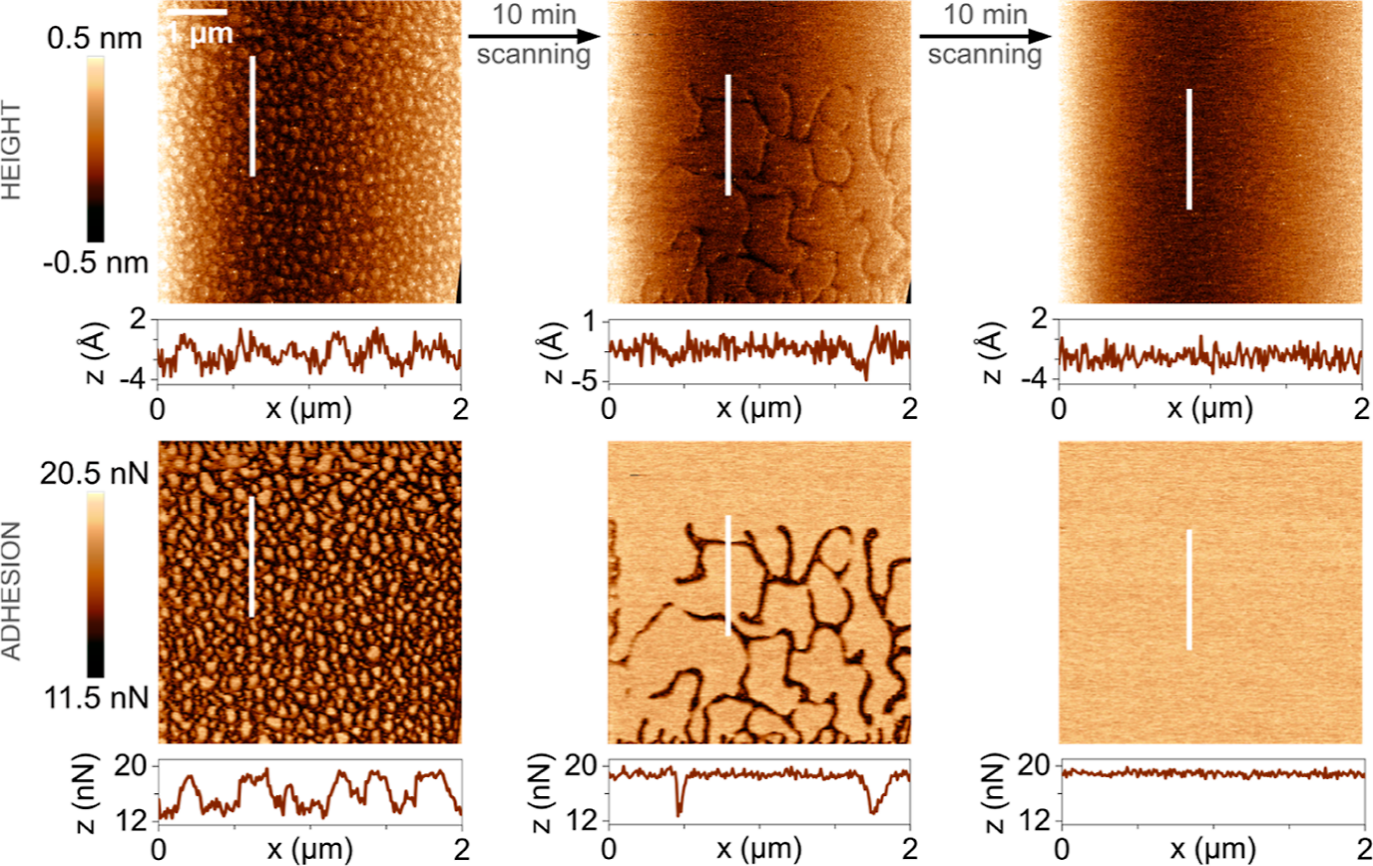
AFM topography and adhesion images of an uncoated freshly cleaved calcite surface. Corresponding deformation images can be found in Figure S1 and Supporting Information. The measurements were done at a RH below 30%.

**Figure 3:**
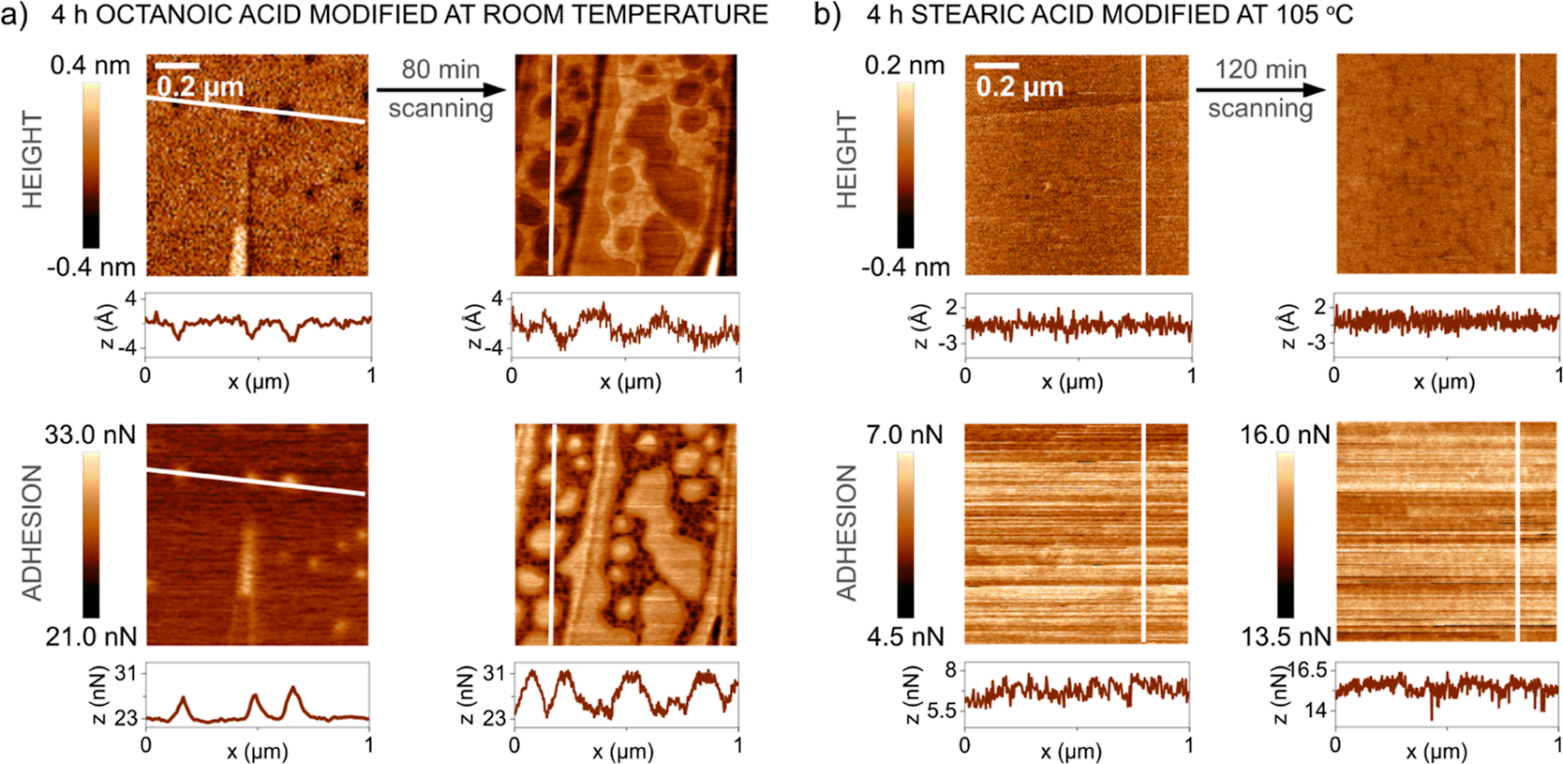
AFM topography and adhesion images of modified calcite surface exposed for 4 h to the vapor of (a) octanoic acid at room temperature (∼25 °C) and (b) stearic acid at 105 °C. Corresponding deformation images can be found in Figure S2 in the Supporting Information.

**Figure 4:**
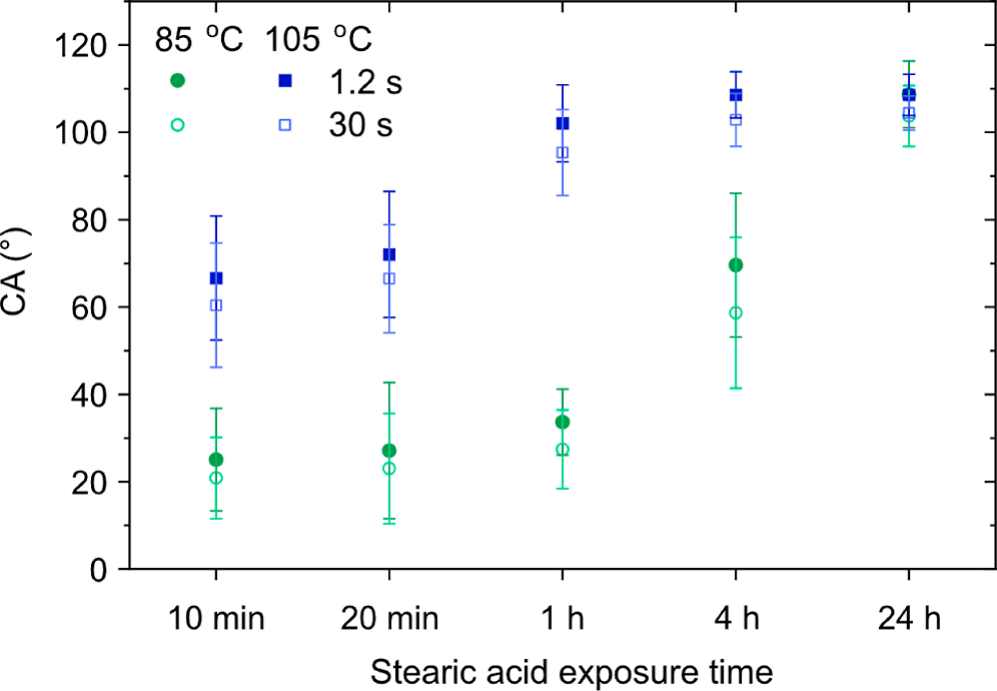
Effect of stearic acid vapor exposure time at 85 and 105 °C on the water contact angles (CA) on surface-modified calcite. Data are presented for two different times (1.2 and 30 s) after droplet deposition. The error bars represent the standard deviation of at least five different measurements.

**Figure 5:**
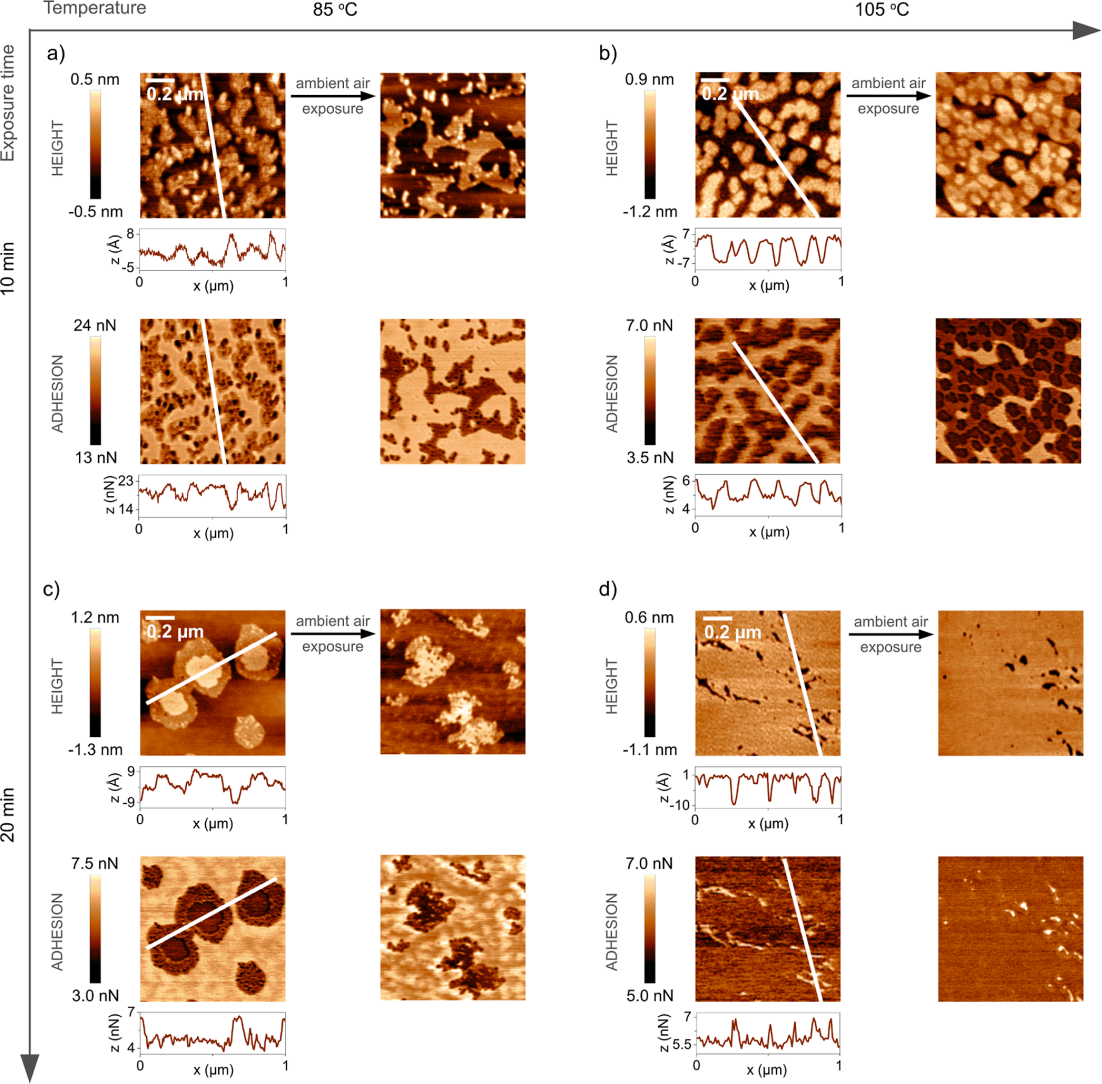
AFM topography and adhesion images of stearic acid on calcite surfaces at different exposure temperatures and times. The stability of the structures during exposure to ambient air (measured a few days after the deposition) is also demonstrated. Stearic acid-modified calcite surface deposited for 10 min at 85 (a) and 105 °C (b) and for 20 min at 85 (c) and 105 °C (d). Corresponding deformation images are presented in Figure S4 in the Supporting Information.

**Figure 6:**
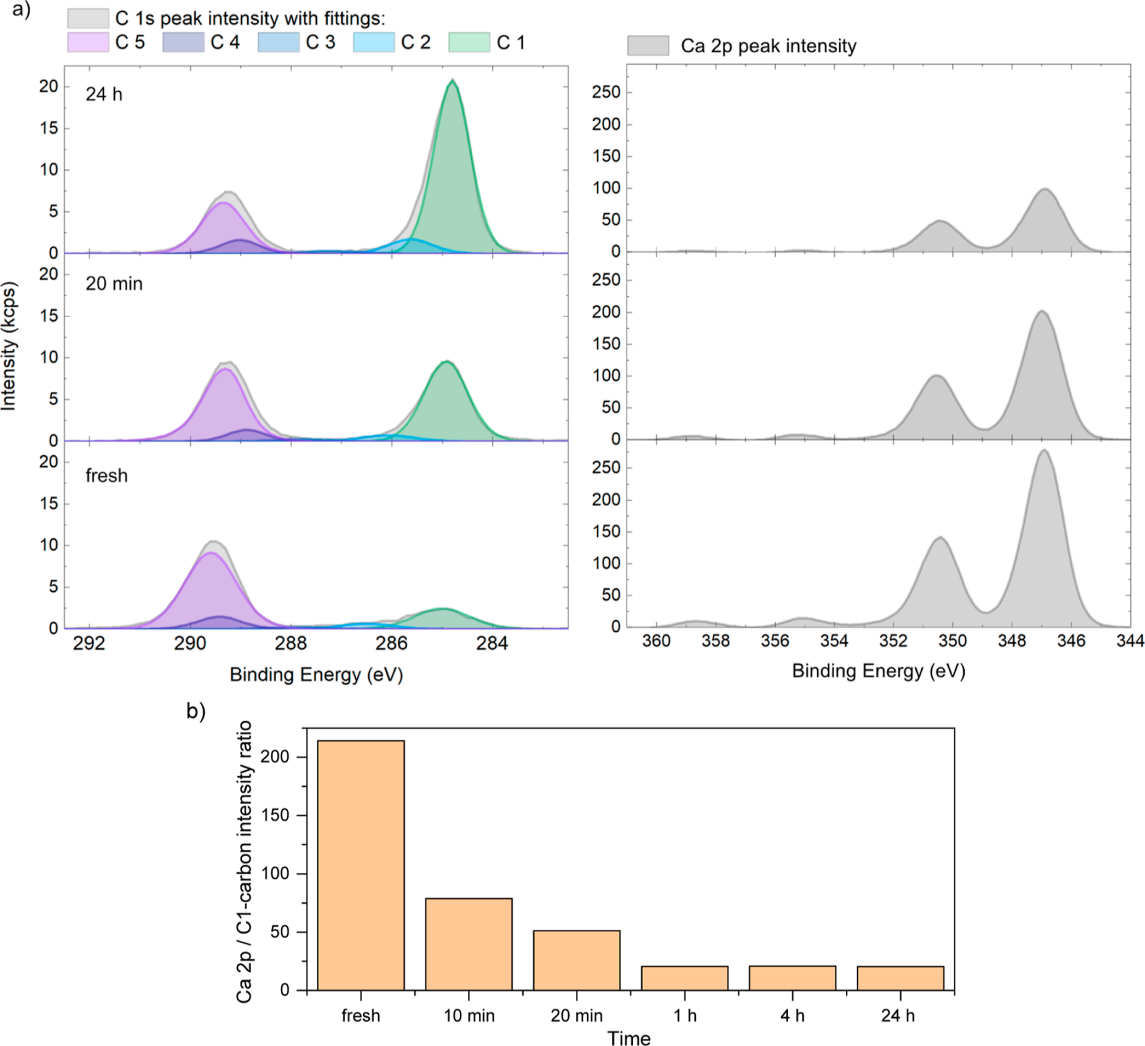
(a) High-resolution XPS spectra of the C 1s and Ca 2p peaks obtained from calcite samples (from bottom: freshly cleaved, 20 min and 24 h exposure to stearic acid vapor at 105 °C). The measured spectra are shown in gray, and the fitted peaks corresponding to C1- to C5-carbons are shown with colors (spectra for additional exposure times can be found in Section 3 of the Supporting Information). (b) Peak intensity ratio Ca 2p/C1-carbon plotted as a function of stearic acid vapor exposure time.

**Figure 7:**
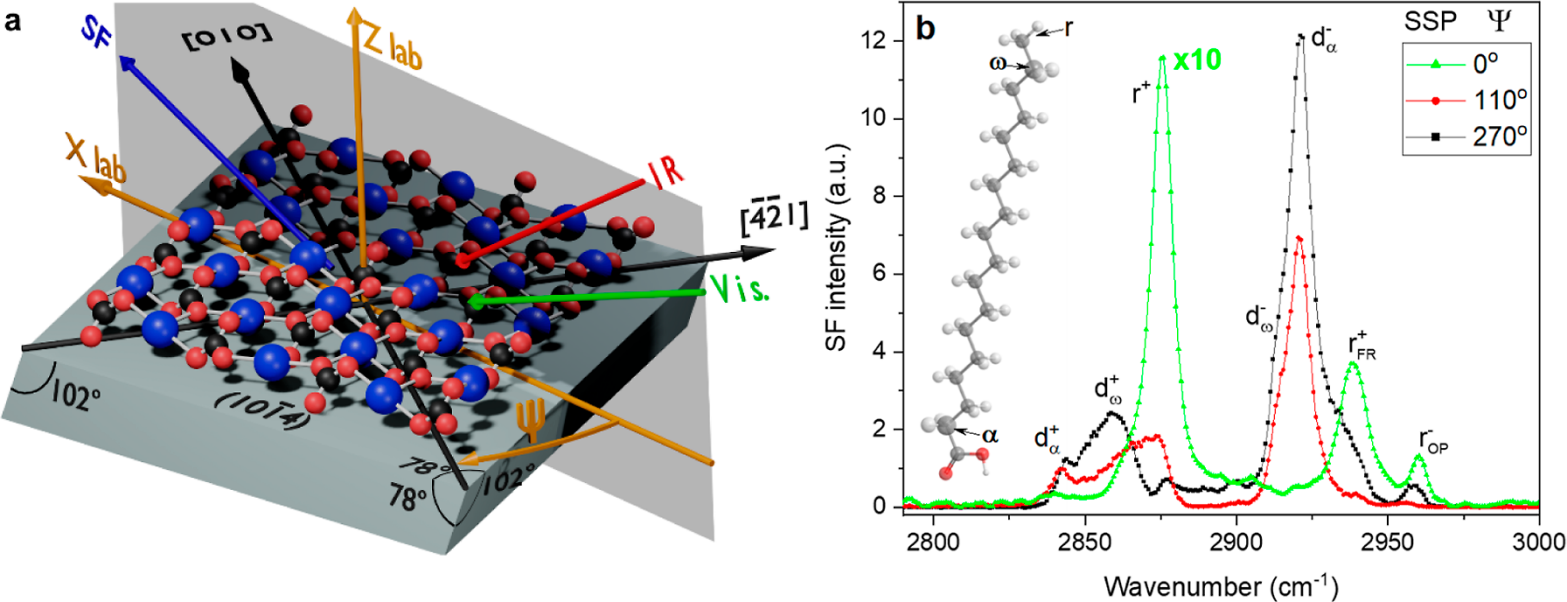
(a) Schematic of the experimental geometry and coordinate system relative to the calcite (101̅4) surface. The red and black spheres depict oxygen and carbon atoms from the carbonate group, and the blue spheres represent calcium atoms. The azimuthal angle (Ψ) is defined between the plane of incidence of the laser beams and the [010] direction, which is perpendicular to the [4̅2̅1] direction and the optical *c* axis. The incident IR and visible laser beams and emitted sum frequency beam are shown in the diagram as red, green, and blue arrows, respectively. *Z*_lab_ corresponds to the axis of rotation in the laboratory frame. (b) Sum frequency spectra of a stearic acid-modified calcite surface, obtained by 24 h exposure to stearic acid vapor at 105 °C, collected under the SSP polarization combination (S-polarized SF beam, S-polarized visible, and P-polarized IR beam) at selected azimuthal angles. Proposed assignments are included in the figure (see the text for details). The spectral region presented corresponds to the CH stretching vibrations from the stearic acid alkyl chain. The molecular model of stearic acid depicts the α and ω CH_2_ groups in the alkyl chain. Note that the spectrum intensity for Ψ = 0° is multiplied by 10 for ease of comparison. Equivalent spectra for 17 additional angles can be found in Section 4 of the Supporting Information (Figure S7).

The VSF spectra were fitted using a convolution of Lorentzian and Gaussian line shapes, which account, respectively, for the homogeneous and inhomogeneous broadening (see Section 4 in the Supporting Information for additional details).^34,35^

## Results and Discussion

The properties of carboxylic acid-modified calcite surfaces were investigated after different carboxylic acid vapor exposure times and exposure temperatures. The coherence and resistance of the formed layers to humid air and water were examined. The studies included measurements of changes in water contact angles for stationary drops as well as advancing and receding contact angles, topography, and nanomechanical mapping by AFM, and evaluation of adsorption and molecular structural properties by means of XPS and VSFS.

### Effect of Carboxylic Acid Chain Length on Surface Wettability Properties

The initial water contact angles (taken after 1.2 s) and the droplet base diameter (BD) determined on modified freshly cleaved calcite surfaces exposed to the vapor of different carboxylic acids for 4 h are shown in Figure 1a. As reported in our previous work,^8^ the water droplet contact angle on freshly cleaved calcite surfaces is low (below 5°) under a nitrogen atmosphere in the absence of any foreign adsorbed molecules. In the current work, after exposure to carboxylic acid vapor, the contact angle was larger showing a strong dependence on the length of the hydrocarbon chain (from 19 ± 7° for acetic acid to 108 ± 5° for stearic acid). The increase in contact angle naturally resulted in a decrease in the base diameter of the droplet from 3.2 ± 0.6 mm for acetic acid to 1.2 ± 0.2 mm for stearic acid. The plots in Figure 1b present the contact angle decrease as a function of time (0 to 30 s) and the associated relative increase in droplet base diameter, , respectively.

On the one hand, for acetic acid (C_2_) and butyric acid (C_4_), both of which are miscible with water, the contact angle and base diameter reached plateau values in only a few seconds, after which an equilibrium between the adsorbed molecules and molecules present in the water droplet seems to be established. On the other hand, for octanoic acid, with a water solubility of 0.8 g/kg (see Table S3 in the Supporting Information), we observed a continuous change in the water contact angle with time. During the 30 s following dispensing of the droplet, the contact angle decreased from 99° to 40° and spread to 170% of the initial base diameter. This molecule has significant air–water surface activity, and the results indicate that equilibration between octanoic acid (C_8_) at the calcite surface, air–water interface, and inside the water droplet was established over a period of time that exceeds the 30 s of the measurements. For the dodecanoic acid (C_12_) and stearic acid (C_18_), with low water solubility (see Table S3 in the Supporting Information), equilibrium is reached at much longer times and the decrease in contact angle and increase in base diameter are modest over the 30 s time period. Certainly, the decreasing water solubility with increasing hydrocarbon chain length resulted in more stable surface layers at these short-exposure times.

### Surface Morphology of Unmodified and Carboxylic Acid-Modified Calcite surface

In order to compare how the changes in the calcite surface morphology are affected by the carboxylic acid vapor exposure, it is crucial to relate them to events occurring on a freshly cleaved calcite surface. The topography and adhesion images obtained by AFM measurements of an unmodified calcite surface when aged in ambient air are presented in Figure 2 (additional AFM images, including those showing deformation properties, can be found in Section 2 of the Supporting Information). As discussed previously,^8^ the calcite surface is not atomically flat due to imperfect cleavage. However, the surfaces analyzed under the AFM probe were selected only on smooth terraces (between the steps and cracks), with an *R*_q_ and *R*_a_ roughness of 0.1–0.2 nm. When exposed to ambient air (30 ± 5% RH), the sample images initially displayed trenches (about 0.3 nm deep) characterized by a lower adhesion and increased deformation compared to the surrounding area (Figure 2 and Section 2 of the Supporting Information). These trenches are likely associated with high stress on the outermost surface caused by the cleaving procedure. In previous spectroscopic studies, it has been shown that dangling bonds generated upon cleavage seek to be satisfied by relaxation, restructuring, or hydration.^36^ Over short exposure time scales, we observed relaxation of the surface structure, leading to the disappearance of the trenches in less than 0.5 h, as seen in Figure 2. Such a process is observed on both heated and unheated freshly cleaved calcite surfaces. We note that it has been suggested that the atomic scale rearrangement on the surface is enhanced by the AFM tip that intermittently contacts the surface with a relatively high force.^36^

Images of modified calcite surfaces after 4 h exposure to carboxylic acid vapor are shown in Figure 3a (octanoic acid) and Figure 3b (stearic acid). In both cases, the surface appeared to be close to fully covered by the carboxylic acids (the visible surface roughness is due to the natural morphology of calcite, such as steps). However, the octanoic acid layer was found to be unstable and relatively easily disrupted by the AFM tip, exposing the more adhesive and less deformable calcite surface underneath (Figure 3a). A similar effect was observed when exposed to ambient air without any AFM scanning, but it happened at a much slower rate (Figure S3 in the Supporting Information). In contrast, the stearic acid layer remained stable with time and during scanning. Thus, the adsorbed stearic acid layer was more stable against mechanical erosion from the AFM tip than that formed by octanoic acid. The fact that increasing the length of the hydrocarbon chain enhances the robustness of the adsorbed layer suggests that van der Waals attraction between the hydrocarbon tails contributes to the layer stability. In our previous work,^8^ we have shown that bare calcite surfaces exposed to ambient air undergo dissolution and recrystallization due to the presence of a thin water layer. No similar changes are observed for stearic acid-modified calcite, suggesting that the hydrophobic character of this layer protects the underlying native calcite surface. In the following sections of this study, we focus our attention on the properties of the stearic acid monolayer.

### Effect of Exposure Time, Temperature, and Ambient Air Storage on Adsorbed Stearic Acid Layer Wettability, Thickness, and Organization

The initial contact angle on stearic acid-modified calcite measured after 1.2 s and the contact angle evaluated after 30 s are compared in Figure 4. Data are shown for different exposure times (10 min to 24 h) to stearic acid vapor at temperatures of 85 and 105 °C. We note that the contact angle increased with increasing exposure time and temperature. For instance, at 85 °C, by extending the exposure time to stearic acid vapor from 10 min to 24 h, the initial contact angle increased from 25 ± 12° up to 109 ± 8°. The data suggest that it takes more than 4 h to reach a coherent layer with a maximum contact angle at 85 °C. In contrast, only about 1 h was sufficient at 105 °C, which is a consequence of the higher vapor pressure at the higher temperature (see Table S2 in the Supporting Information).

It is generally accepted that it is the carboxylic acid functional group that preferentially binds to the calcite surface, which eventually leads to monolayer formation.^4,23^ However, it is less clear how this layer builds up, and we now turn our attention to this question by collecting AFM images after different adsorption times. With increasing time in the stearic acid vapor environment, new domains with increased height appeared on the surface, as shown in Figure 5. Under the compression of the AFM tip, we find a height of 1.4 nm (about 56% surface coverage) after exposure to stearic acid vapor at 105 °C for 10 min (Figure 5b), and a combination of heights of 0.7 nm (about 52% coverage) and 1.4 nm (about 58% coverage) after exposure at 85 °C for 10 and 20 min, respectively (Figure 5a,c). Thus, it is clear that initially, stearic acid was not uniformly distributed over the surface, but domains with a high density of stearic acids were formed. These domains grew with time and eventually merged into a complete monolayer, as seen in Figures 5d and 3b. The fact that domains formed demonstrates that it was energetically more favorable for an arriving molecule to attach next to an already adsorbed stearic acid molecule than in a location with no stearic acid neighbors. Thus, there is a favorable interaction between the adsorbed molecules, which we attribute to attractive van der Waals forces between the hydrocarbon chains. The growth of the domains and their merging to a complete monolayer occurred more rapidly at higher temperatures, which is consistent with the wettability data, as reported in Figure 4. Over the exposure time, the compressed height of the stearic acid domains found at 85 °C homogenized toward 1.4 nm, until full coverage was reached, and the height differences naturally disappeared. The reduced adhesion and increased deformation compared with the calcite substrate are due to the presence of the soft stearic acid layer. The variation in adhesion force across the images can be attributed to the use of different probes (yet, of the same type). By adding the deformation difference (data shown in Section 2 of the Supporting Information) between the stearic acid domain areas and the bare calcite surface to the compressed layer thickness, we obtain a value of the height for the undisturbed stearic acid domain of ∼1.9 nm (as a comparison, the extended length of stearic acid is 2.4–2.6 nm^4,37^).

A crucial aspect of calcite surface modification is the resistance of the adsorbed layer to ambient air conditions. As presented further in Figure 5a–c, patchy stearic acid layers were not stable in humid air. We found changes in the stearic acid domain structure as well as in the unmodified calcite substrate areas over long exposure times. This was related to the surface dissolution and recrystallization process that occurs on freshly cleaved calcite surfaces aged in ambient air (see Figure S1 in the Supporting Information). Importantly, calcite surface reorganization was not observed when stearic acid almost fully covered the surface (after at least 20 min of exposure to C_18_ at 105 °C, Figure 5d), which appeared to block water from reaching the calcite interface.

In summary, stearic acid initially formed domains on the calcite surface, which with time grew to a homogeneous, interconnected layer. The faster monolayer formation at increased temperatures was due to the corresponding higher vapor pressures, thus resulting in more stearic acid molecules reaching the surface per unit time. A higher temperature is also expected to increase the reorganization rate in the layer, as well as the rate of possible chemical reactions with the calcite surface. Full coverage of stearic acid in the form of a monolayer counteracted the surface dissolution and recrystallization processes found for unmodified calcite surfaces in contact with ambient air.

### Stearic Acid Adsorption on Calcite Surfaces Probed by XPS

Our quantification of the adsorption of stearic acid on calcite by means of XPS relies on accurate measurements of the Ca 2p and C 1s peaks originating from both the substrate (C5-carbon peak, carbonate) and the adsorbed stearic acid layer (C1-carbon peak). The C 1s peak is deconvoluted into five different carbon peaks (i.e., C1- to C5-carbon), corresponding to different oxidation states of the carbon atom, as detailed in Section 3 of the Supporting Information. Examples of such deconvoluted spectra for a freshly cleaved calcite surface and for calcite surfaces modified by stearic acid at selected acid vapor exposure times are shown in Figure 6a.

In the theoretical chemical structure for calcite, only carbon atoms in the form of a C5 carbon (carbonate) are present. For the freshly cleaved calcite surface in Figure 6a, indeed, the strongest peak is the C5- carbon peak at a binding energy just below 290 eV. However, in addition, weaker peaks of C1–C4- carbons (from organic carbons) are observed at lower binding energies. The total amounts of C1–C4- carbons detected for freshly cleaved calcite were found to be between 8% and 10 at. %, with the major part from C1-carbon. This level of organic carbon is common to find on any air-exposed surface, such as freshly cleaved minerals, and it originates from airborne adventitious carbon contamination.

For calcite surfaces modified by stearic acid, the C 1s signal changes characteristics, as shown in Figure 6a. With a longer exposure time, the C1-carbon peak contribution to the C 1s envelope becomes more apparent. This peak is specific to the hydrocarbon chain of stearic acid and indicates an increase in the adsorbed amount. At the same time, the C5-carbon component from the underlying calcite substrate decreased in intensity due to the shielding effect from the adsorbing stearic acid overlayer (photoelectron absorption in the adsorbed layer). For the same reason, the intensity of the Ca 2p peak decreased with increasing exposure time to stearic acid vapor (Figure 6a). Finally, we note that a plateau in the intensity ratio Ca 2p/C1-carbon is reached after about 1 h, demonstrating that no further adsorption of stearic acid occurs beyond this point.

#### Determination of Layer thickness

The thickness of the adsorbed stearic acid layer was determined from measurements at different takeoff angles (see data in Section 3 of the Supporting Information). In contrast to the Ca 2p signal that exclusively originates from the calcite substrate, the O 1s signal also includes a contribution from oxygens in the carboxylic/carboxylate group in the adsorbed stearic acid monolayer. Consequently, only the Ca 2p signal is used for further analysis (eq S2 in the Supporting Information). From the slope of the linear fit to the data, the reduced thickness *t*_o_/λ_a=Ca2p_^°^ was determined, where *t*_0_ is the adsorbed (overlayer) layer thickness, and λ_Ca2p_^°^ is the inelastic mean free path (IMFP) in the stearic acid layer for photoelectrons emitted from Ca 2p orbitals in the substrate. The λ_Ca2p_^°^ value was calculated to be 3.6 nm following the method of Cumpson,^38^ while for the overlayer element d, λ_d=*C*1*s*_^°^ was determined to be 3.7 nm. Both these IMFP values are consistent with other reports.^39^ The calculated stearic acid overlayer thicknesses after different exposure times are reported in Table 1.

**Table 1:** Stearic Acid Layer Thickness, Adsorbed Stearic Acid Molecules Per Unit Area, and Area Per Molecule as a Function of Exposure Time to Stearic Acid Vapor at 105 °C

exposure time (h)	layer thickness (nm)	lower limit		upper limit	
		molecules/area (1/nm^2^)	area/molecule (nm^2^)	molecules/area (1/nm^2^)	area/molecule (nm^2^)
1	2.7	5.2	0.19	6.2	0.16
4	2.8	4.9	0.20	5.9	0.17
24	2.9	5.3	0.19	6.3	0.16

We note that the XPS analysis and substrate-overlayer model assumed in our calculations is valid for a homogeneous fully covering overlayer on a substrate surface, as observed with AFM on surfaces exposed to the stearic acid vapor for 1 to 24 h (Figure S5 in the Supporting Information). This is the reason why angular-dependent studies were not performed for exposure times shorter than 1 h. The thickness of the stearic acid layer determined by XPS, 2.7–2.9 nm, was slightly larger than that of an extended stearic acid monolayer (2.4–2.6 nm^4,37^). This could suggest that some stearic acid molecules were physisorbed on top of the monolayer or that some of the original adventitious carbon contamination found on the freshly cleaved calcite surface remained after stearic acid adsorption. We note that the former option is not supported by the VSFS results presented below. With AFM, we obtained an undisturbed thickness of up to ∼1.9 nm of the patchy layer obtained at short exposure times (20 min or shorter). The layer thickness is expected to increase as the adsorption proceeds during prolonged exposure to stearic acid vapor, and we conclude that AFM and XPS data compare favorably.

#### Determination of Adsorbed Amount

The stearic acid adsorbed amount is determined from the Ca 2p and the C1-carbon peaks, following a procedure described in detail in Section 3 of the Supporting Information (eq S3). The analysis is complicated by the fact that a C1-carbon peak is also observed on freshly cleaved calcite due to adventitious carbon contamination, and the fate of these CH-containing molecules upon the adsorption of stearic acid is not known.

The C1-carbon present on the freshly cleaved calcite surface could, in principle, be either entirely, partially, or not removed at all upon the adsorption of stearic acid. For this reason, we carried out the calculations in two ways, by assuming either (i) that none of the C1-carbon found on freshly cleaved calcite was displaced by the stearic acid or (ii) that all C1-carbon found on the freshly cleaved calcite surface was displaced. The true result is most likely somewhere between these two cases.

The data of the upper and lower limits in adsorbed amount of stearic acid calculated using the two assumptions mentioned above are displayed in Table 1. The lower limit was obtained by subtracting the C1-carbon signal found on freshly cleaved bare calcite from the C1-carbon signal found for stearic acid-modified calcite. The upper limit was calculated without any such subtraction. As expected, the adsorbed amount remained close to constant in the exposure time interval 1 to 24 h. We note that the area per molecule for the lower limit case was close to that for a tightly packed fatty acid monolayer (∼0.20 nm^2^^40,41^) with the hydrocarbon chains in the all-trans conformation. The upper limit case suggests adsorption of slightly more than a monolayer. Thus, despite some uncertainty in the adsorbed amount, we can conclude that stearic acid forms a tightly packed layer of about one monolayer thick on calcite when exposed to stearic acid vapor for at least 1 h at 105 °C.

### Characterization of the Adsorbed Stearic Acid Monolayer Using Vibrational Sum Frequency Spectroscopy

VSFS has an exquisite sensitivity to conformational order of organic monolayers,^42^ and can also provide direct information on how the fatty acid headgroup interacts with the calcite surface. With its intrinsic surface selectivity, the sum frequency (SF) response is typically free from bulk contributions that usually overlaps with the features of interest in the IR spectra of equivalent long alkyl chain monolayers on calcite.^4^ The SF spectra of stearic acid adsorbed on the calcite surface after a 24 h exposure displayed a strong dependence on the azimuthal angle orientation of the substrate. This is shown in the data presented in Figure 7b for the selected rotational angles collected under the SSP polarization combination (the three letters referring to the polarization of the SF, visible, and IR beams, respectively). Spectra for the full set of all angles measured can be found in Section 4 of the Supporting Information and Figure S7. The azimuthal angle (Ψ) is defined as the angle between the plane of the incident laser beams and the crystallographic [010] direction on the (101̅4) plane, as illustrated in Figure 7a. The fact that the spectra change with Ψ indicates that the monolayer is not isotropic in the plane of the surface. The adopted structure is then dictated by interactions with the calcite substrate. We note that consistent with this conclusion, SF intensity could be detected in polarization combinations (i.e., SSS, SPP, PSP, and PPS) that are otherwise forbidden for monolayers that are isotropic in the plane (see selected spectra in Figure S8 in Section 4 of the Supporting Information). Moreover, the spectral features and their azimuthal angle dependence were remarkably similar between different locations within the same sample as well as between different calcite samples, implying that the two-dimensional preferential order in the epitaxial monolayer is highly reproducible and extends throughout the plane of the surface.

The spectral range presented in Figure 7b corresponds to the CH stretching modes of the alkyl chain of the stearic acid monolayer. The bands observed in the Ψ = 0° spectrum are all associated with the terminal methyl group, mainly the symmetric stretch (r^+^) at ∼2873 cm^–1^, its Fermi resonance at ∼2940 cm^–1^ (r^+^_FR_), and the out of phase antisymmetric stretch (r^–^_OP_) at ∼2960 cm^–1^. In VSFS, the lack of features linked to methylene groups in the chain is indicative of conformationally ordered monolayers in an all-trans configuration.^43−45^ However, upon azimuthal rotation of the sample, new bands associated with methylene groups become apparent and dominate the spectra. Two distinct symmetric methylene stretches at 2842 and 2865 cm^–1^ can be identified in the Ψ = 110° and Ψ = 270° spectra (see Figure 7b). The latter has been assigned to the terminal methylene group (*d*^+^_ω_) adjacent to the CH_3_ in the hydrocarbon chain,^46,47^ while the former we assign to the methylene group in the α carbon next to the carboxylic acid headgroup (*d*^+^_α_). The assignments are supported by the AFM and XPS results discussed above, as well as previous IR^4^ and X-ray reflectivity^37^ studies on stearic acid-modified calcite surfaces prepared using a solvent-based approach, which conclude that stearic acid forms tightly packed monolayers in an all-trans configuration with the chains almost perpendicularly oriented to the surface, i.e., along the surface normal. Consequently, the presence of CH_2_ vibrations in the SF spectra cannot be associated with extensive *gauche* defects in the alkyl chain but originates instead from the terminal chain locations where the symmetry is necessarily broken, and the methylene vibrational modes are decoupled from those within the chain.^45^ In the spectra shown in Figure 7b, two stronger bands also linked to the methylene groups are observed at higher frequencies. They are the corresponding antisymmetric stretches centered at ∼2912 cm^–1^ (d^–^_ω_) and 2921 cm^–1^ (d^–^_α_). The intensity of these bands varies from essentially zero at Ψ = 0° to approximately ten times that of the r^+^ peak at Ψ = 90° and 270° (note that the amplitude of the Ψ = 0° spectrum in Figure 7b is multiplied by ten for ease of comparison). In surfaces that are isotropic in the plane, antisymmetric methylene vibrations are usually weak^45^ and, in the SSP polarization, always less intense than the associated symmetric modes.^32^ Clearly, the rotation anisotropy has notable consequences in SF spectra. Similar epitaxial effects have previously been observed on a Langmuir–Blodgett transferred zinc arachidate monolayer on an alumina substrate.^48^

**Figure 8:**
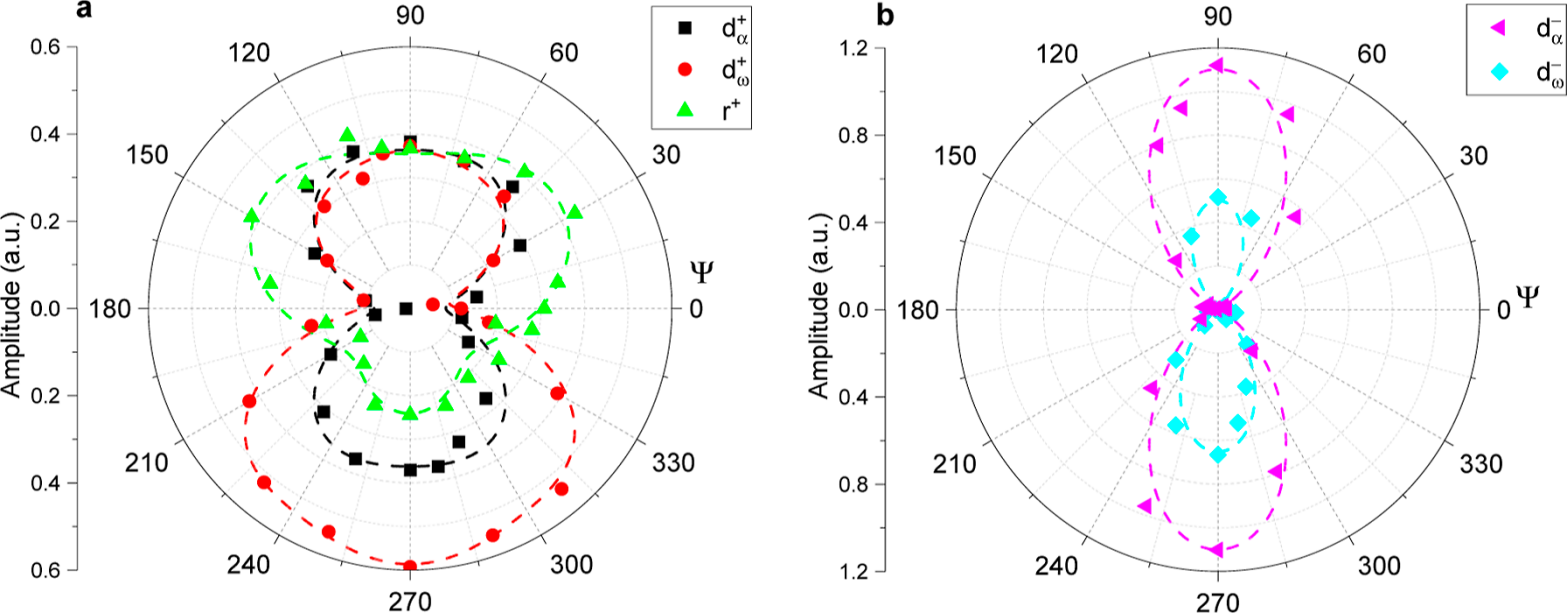
Polar plots showing the azimuthal dependence of the fitted amplitudes of (a) symmetric CH_3_ (r^+^), α-CH_2_ (d^+^_α_), and ω-CH_2_ (d^+^_ω_), and (b) antisymmetric α-CH_2_ (d^–^_α_), and ω-CH_2_ (d^–^_ω_) stretching modes. The in plane rotation angle (Ψ) changes counterclockwise from 0 to 360°. The experimental data are depicted as points, and the fits to the azimuthal dependence are depicted as segmented lines. All fitted parameters can be found in Section 4 of the Supporting Information.

The azimuthal dependence of each vibrational mode is best appreciated in polar plots that show variations of the fitted amplitudes as a function of angle Ψ. These are presented in Figure 8a,8b for the CH_2_/CH_3_ symmetric and antisymmetric CH_2_ stretching modes, respectively. The azimuthal dependence for each vibrational mode is also fitted to a set of trigonometric functions to help visualize the changes (see the short segment lines in Figure 8). Twofold symmetric patterns can be readily identified for the methylene modes. However, they are symmetric for the α-CH_2_ (d^+^_α_ and d^–^_α_), and asymmetric for the CH_2_ group next to the terminal methyl group (d^+^_ω_ and d^–^_ω_). Moreover, the fitted amplitudes have also different signs (see fitted values in Table S4 in the Supporting Information), which implies that the two methylene groups have opposite net polar orientations.^32,49^ An orientational analysis can be performed to determine the corresponding molecular twist and tilt angles that are the most consistent with the measured azimuthal angle patterns.^50−52^ However, this topic will be the subject of a separate study.

### Headgroup Vibrations

**Figure 9:**
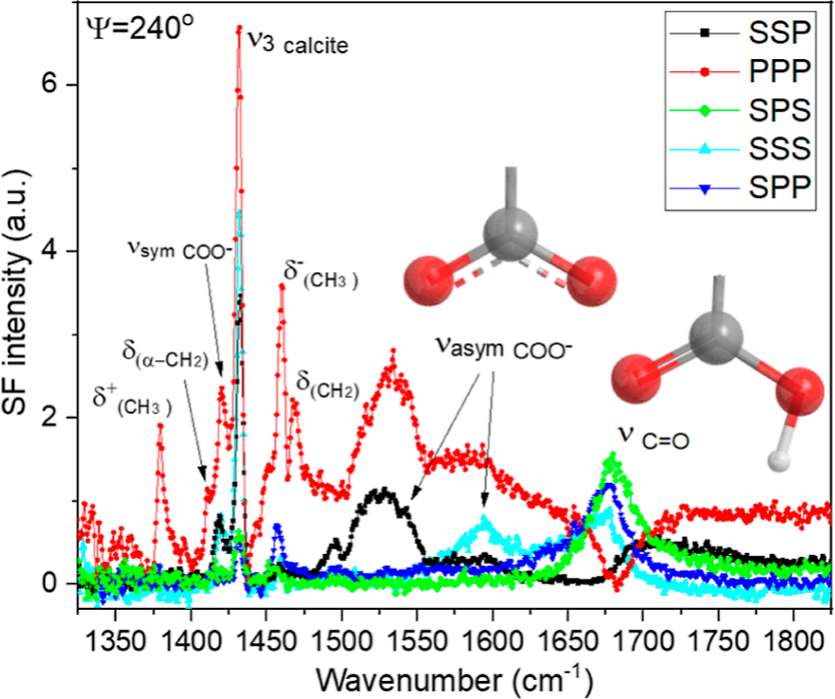
Sum frequency spectra of a 24 h stearic acid-modified calcite surface in the double-bond/fingerprint region collected under the SSP, PPP, SPS, SSS, and SPP polarization combinations at Ψ = 240° (the three letters refer to the polarization of the SF, visible, and IR beams, respectively). Proposed assignments and sketches of the carboxylic and carboxylate groups are included in the figure (see the text for details).

Additional insights into the interactions of stearic acid with the calcite surface can be obtained by targeting molecular vibrations from the carboxylic acid headgroup. Figure 9 shows the SF spectra in the double bond/fingerprint region of a stearic acid monolayer on calcite measured at five different polarization combinations (Ψ = 240°). As expected, rotational anisotropy is also observed in this spectral region; i.e., SF signal is detected in polarizations that are “forbidden” in isotropic surfaces. Vibrational modes from the carboxylic acid headgroup, as well as those from the alkyl chain and the underlying calcite surface, overlap in the spectra. The most intense peak centered at 1432 cm^–1^ is assigned to the antisymmetric carbonate stretch from the calcite surface (*n*_3calcite_),^53^ as it is seen in the SF spectra of a freshly cleaved unmodified calcite sample (see Figure S9 in the Supporting Information). There are also several bands linked to the bending and deformation modes from the methyl and methylene groups. They include the symmetric (δ^+^_CH3_) and asymmetric (δ^–^_CH3_) methyl deformations at 1380 and 1460 cm^–1^, respectively,^41,54^ and the methylene deformation (δ_CH2_) at 1469 cm^–1^. Note that due to headgroup proximity, the deformation vibration for the methylene group next to carboxylic acid (δ_α-CH2_) is observed at lower frequencies, ∼1410 cm^–1^.^41,55^ Most importantly, the vibrational modes from both the protonated and deprotonated forms of the carboxylic acid moiety can be resolved in the spectra. Evidence for the protonated (i.e., uncharged) case comes from the peak centered at 1680 cm^–1^, assigned to the carbonyl stretch (ν_C=O_) of the COOH group. The band is on calcite ∼40 cm^–1^ red-shifted when compared to that in Langmuir monolayers where the carbonyl is in contact with water.^41,56^ The shift implies a weakening of the double bond strength, which can be explained by direct electrostatic interactions between the carbonyl and calcium atoms on the calcite surface. This is consistent with the MD simulation of a shorter chain carboxylic acid adsorbed onto CaCO_3_, where the carboxylic acid was constrained to remain protonated.^57^ On the other hand, the sum frequency spectra also show direct evidence for the deprotonated form, primarily through the asymmetric carboxylate stretches centered at ∼1545 and ∼1590 cm^–1^, but also the symmetric carboxylate stretch at ∼1420 cm^–1^.^58,59^ Consequently, the headgroup of the vapor deposited stearic acid can be found in both deprotonated (formation of stronger COO^–^·· calcium contact ion pairs), and protonated forms (i.e., C=O···calcium ionic interaction).

## Conclusions

The vapor deposition method was successfully employed to create both patchy and uniform carboxylic acid layers on calcite under well-controlled conditions. This approach sheds light on the progression of the layer formation: initially, the fatty carboxylic acids form patches on the calcite surface due to attractive van der Waals interactions between the hydrocarbon chains. As vapor deposition continues, these domains merge and form a continuous layer. The process was followed by AFM measurements of topography and nanomechanical properties, where carboxylic acid patches were distinguished by their lower adhesion and larger deformation compared to those of the untreated bare calcite.

The studies considered the impact of the length of the carboxylic acid alkyl chain (ranging from C_2_ to C_18_), vapor exposure time (from 10 min to 24 h), and deposition temperature (25 °C up to 105 °C) on the properties of the adsorbed layer. As the packing density and the chain length of the carboxylic acid increased, the modified calcite surface became more hydrophobic, and the stability of the layer when exposed to air and water droplets also increased. The latter observation is primarily attributed to the lower solubility of carboxylic acids in water when increasing the hydrocarbon chain length. Further details concerning structural changes in the stearic acid layer due to contact with water droplets can be found in our recent work.^25^

The coherent and full-covering stearic acid layer was analyzed in detail in terms of the adsorbed amount and thickness using XPS. The amount of stearic acid adsorbed onto the calcite surface reaches saturation at a level corresponding to monolayer coverage. Within the uncertainties imposed by the likely presence of adventitious hydrocarbon contaminants on freshly cleaved calcite before the surface modification, the independently determined thickness is also consistent with that expected for a monolayer.

Novel insights into the arrangement of the coherent monolayer of stearic acid on calcite were obtained by using VSFS. The spectra confirmed that the hydrocarbon chains adopt an all-trans configuration. Notably, the data revealed that the layer had an anisotropic orientation within the plane of the calcite surface, indicating a two-dimensional preferential order in the epitaxial monolayer that spans the entire surface. The same orientational order was observed across multiple spots and on different surfaces. Additionally, the vibrational spectra unambiguous show the presence of both the carboxylate and carboxylic acid forms of stearic acid within the adsorbed layer. The protonated form was identified through the C=O vibration, which displayed a significant red shift due to interactions with calcium ions at the calcite surface. The stearate form was detected via the asymmetric carboxylate stretches. Hence, the layer formed by vapor deposition contains both uncharged (protonated) and charged (deprotonated) forms of stearic acid.

## Supporting Information Available

Vapor pressure and aqueous solubility of carboxylic acids, AFM deformation, and morphology of carboxylic acid-modified calcite surfaces, XPS adsorption and thickness calculations, and additional VSFS spectra and fitting parameters (PDF)
