# Supplementary material for: Calcite Surfaces Modified with Carboxylic Acids (C2 to C18): Layer Organization, Wettability, Stability, and Molecular Structural Properties
Source: Langmuir. 2023 Oct 12;39(42):14840–52. doi: 10.1021/acs.langmuir.3c01252 (PMC10601537; doi:10.1021/acs.langmuir.3c01252)
Supplement: Supplementary file 1 — la3c01252_si_001.pdf [file la3c01252_si_001.pdf]

Supporting Information

Calcite surfaces modified with carboxylic acids (C_2 to C_{18}): layer organization, wettability, stability, and molecular structural properties.

Natalia A. Wojas [*,a,b] Eric Tyrode[*,b] Robert Corkery [b,c] Marie Ernstsson [a] Viveca Wallqvist [a] Mikael Järn [a] Agne Swerin [d] Joachim Schoelkopf [e] Patrick A. C. Gane [f,g] Per M. Claesson [*,b]

[a] RISE Research Institutes of Sweden, Division of Bioeconomy and Health - Material and Surface Design, Box 5607, SE-114 86 Stockholm, Sweden

[b] KTH Royal Institute of Technology, Department of Chemistry, Teknikringen 30, SE-11428, Stockholm, Sweden

[c] Australian National University, Department of Applied Mathematics, Research School of Physics and Engineering, Canberra, ACT 0200, Australia

[d] Karlstad University, Faculty of Health, Science and Technology, Department of Engineering and Chemical Sciences: Chemical Engineering, SE-651 88 Karlstad, Sweden

[e] Omya International AG, Baslerstrasse 42, CH-4665 Oftringen, Switzerland

[f] Aalto University, School of Chemical Engineering, Department of Bioproducts and Biosystems, P.O. Box 16300, FI-00076 Aalto, Finland

[g] University of Belgrade, Faculty of Technology and Metallurgy, Karnegijeva 4, 11200 Belgrade, Serbia

***Corresponding authors:** natalia.anna.wojas@ri.se, tyrode@kth.se, and percl@kth.se

Content:

		Pages
**1.**	Vapour pressure and aqueous solubility of carboxylic acids	S2-S3
**2.**	AFM: Surface morphology of unmodified and carboxylic acid modified calcite surface	S4-S5
**3.**	XPS calculations	S6-S8
**4.**	Vibrational Sum Frequency Spectroscopy	S9-S11

1. Vapour pressure and aqueous solubility of carboxylic acids

Surface modification via carboxylic acid vapour exposure is efficient only at high enough vapour pressure. The vapour pressures were estimated from the Antoine equation or the reported vaporization enthalpies and their temperature dependence.[1-2] For the latter case, a third-order polynomial fit of the form shown in **Eq. S1** was used:

$$\ln\left(\frac{p}{p_{atm}}\right)_{calculated} = A\,T^{-3} + B\,T^{-2} + C\,T^{-1} + D \qquad \textbf{(S1)}$$

where p is the vapour pressure, T the temperature (K), and A, B, C and D fitted constants summarized in **Table S1**.[1] The vapour pressures of the different fatty acids used under our experimental conditions are presented in **Table S2**.

Table S1. Coefficients of Eq. S1 for stearic acid (valid up to the boiling temperature: 648.1 K)

**Acid**	**A**	**B**	**C**	**D**
Stearic acid	5.17E8	-5.69E6	5.47E3	3.27

Table S2. Vapour pressure for water and carboxylic acids (C_2-C_8 [3], C_{12} [4], C_{18}[1]). Liquid state of the media is marked as (^{liq}).

**Medium**	**Vapor pressure (Pa) at temperature:**				
	**25 °C**	**60 °C**	**80 °C**	**85 °C**	**105 °C**
water	3173 ^{liq}	-	-	-	-
C_2	2093 ^{liq}	-	-	-	-
C_4	220 ^{liq}	-	-	-	-
C_8	0.49 ^{liq}	-	-	-	-
C_{12}	$2.1 \cdot 10^{-3,*}$	77 ^{liq,*}	512 ^{liq,*}	-	-
C_{18}	$9.6 \cdot 10^{-5}$	-	-	0.134 ^{liq}	1.53 ^{liq}

* Estimated for temperatures outside the valid range of the Antoine equation

The melting point and aqueous solubility of the carboxylic acids used in the present work are provided in **Table S3**.

Table S3
Normal melting point of carboxylic acids and their aqueous solubility in grams per kilogram of water at 21-23 °C. The mark ∞ means that the carboxylic acid is completely miscible with water [3].

Acid	Melting point (°C)	Aqueous solubility (g/kg)
C_2	17.3	∞
C_4	-5.1	∞
C_8	16.5	0.800
C_{12}	43.8	0.060
C_{18}	69.3	0.003

2. AFM: Surface morphology of unmodified and carboxylic acid modified calcite surface

Figures S1 and **S2** show deformation images of bare calcite and calcite modified by carboxylic acids, respectively. These images are recorded at the same time as the topography and adhesion images reported in **Figure 2** and **Figure 3**, respectively, in the main text.

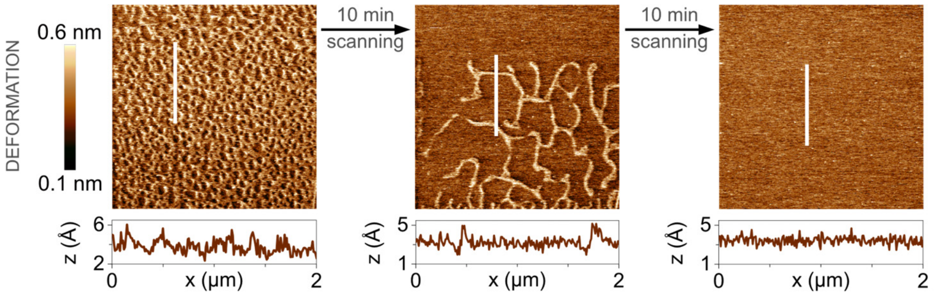

Figure S1. AFM deformation images of an uncoated freshly cleaved calcite surface (similar behaviour to that of a freshly cleaved calcite heated for 20 min at 85 °C.

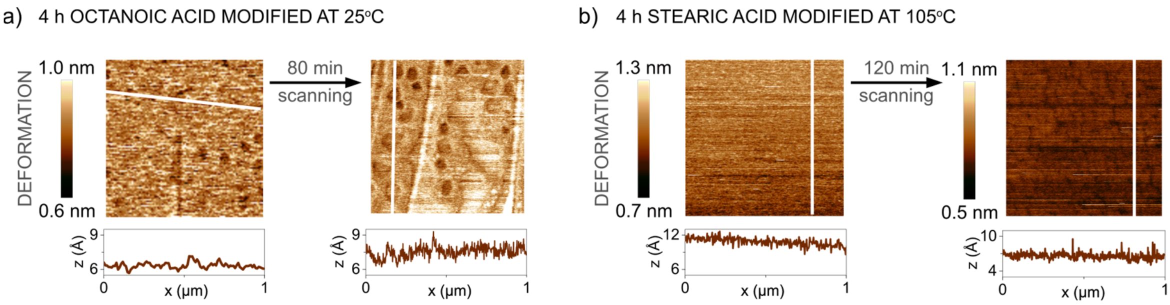

Figure S2. AFM deformation images of modified calcite surface exposed for 4 h in (a) octanoic acid vapour at room temperature (25 °C), and (b) stearic acid vapour at 105 °C

A calcite surface containing an octanoic acid layer rearranges with time when exposed to ambient air. As compared to immediately after preparation (**Figure 3** in the main text), the surface is less homogeneous.

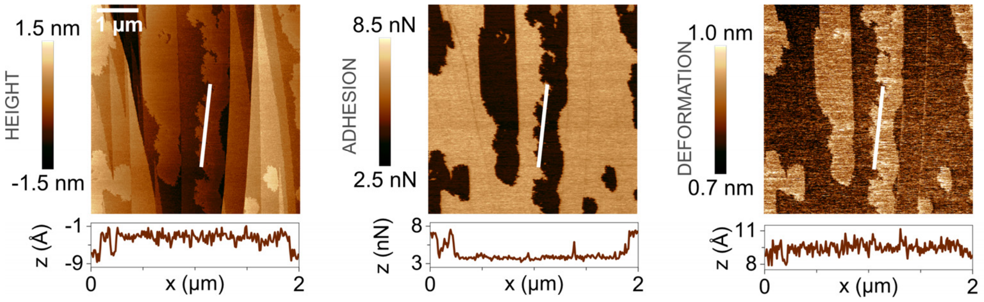

Figure S3. AFM topography and nanomechanical mapping images of modified calcite surface exposed for 4 h in C_8 vapour at room temperature (~25 °C) and stored overnight in ambient air.

Deformation images recorded at the same time as the topography and adhesion images reported in **Figure 5** of the main text are reported in **Figure S4**:

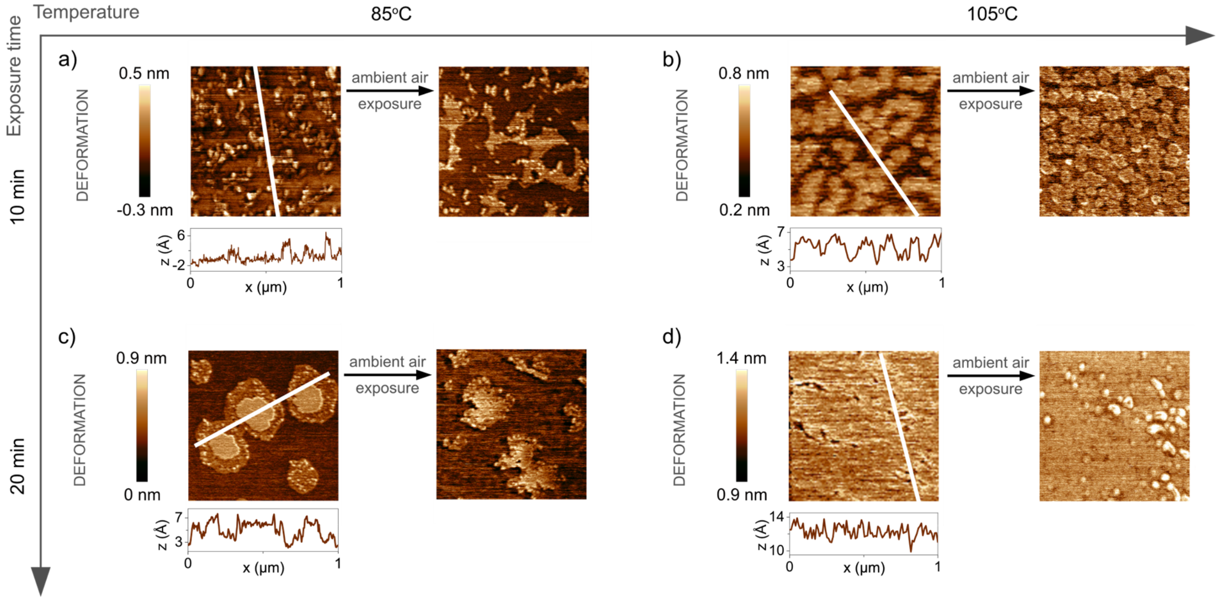

Figure S4. AFM deformation images of stearic acid organization on calcite surfaces at different exposure temperatures and times, as well as their stability over longer exposure to ambient air (measured a few days after deposition). Stearic acid modified calcite surface deposited for **(a)** 10 min at 85 °C, **(b)** 10 min at 105 °C, **(c)** 20 min at 85 °C, and **(d)** 20 min at 105 °C.

3. XPS calculations

The relative amounts of carbon species with different bonds to oxygen were determined from the high-resolution carbon C 1s spectra by deconvolution into five different carbon peaks using the Gaussian – Lorentzian peak shape model. We considered five different chemical environments for the carbon atom. C1-carbon at 285.0 eV (C-C, C-H functional groups), C2-carbon at 286.4 – 286.7 eV (C-O, C-O-C), C3-carbon at 287.9 – 288.1 eV (C=O, O-C-O), C4-carbon at 289.2 – 289.4 eV (O-C=O, C(=O)OH) and C5-carbon at 289.6 – 289.9 eV (carbonate).[5] Clearly, as also shown in **Figure 6a**, the C4- and C5-carbon peaks overlap and are not readily resolved into two separate peaks. For this reason, the theoretical atomic ratio Ca / C5-carbon = 1 in calcite was used to calculate the size of the C5-carbon peak. The remaining part in the broad C4 – C5 carbon peak is then the smaller C4 carbon peak.

The adsorbed layer, overlayer, thickness t_o, was calculated under the assumption that a uniform stearic acid layer is present on a homogeneous calcite substrate, as for the substate-overlayer model presented in **Figure S5**. In this case, the photoelectron peak intensity from element *a* in the substrate, I_a, (area of the peak after background subtraction, see **Figure 6** in the main text) is given by [6]:

$$I_a = S_a n_a e^{\left(-\frac{t_o}{\lambda_a^o \sin\theta}\right)} \quad \textbf{(S2)}$$

where S_a is the empirically derived atomic sensitivity factor (also known as relative sensitivity factor RSF) for element a, n_a the volume atomic density of element a, λ_a^o the photoelectron inelastic mean free path (IMFP) in the stearic acid overlayer for photoelectrons emitted from element *a* in the substrate, and θ the photoelectron take-off angle. The reduced thickness, t_o/λ_a^o, was determined from **Eq. S2** by plotting the natural logarithm of the substrate signal intensity I_a (where a is the substrate signal Ca 2p) as a function of $-1/\sin\theta$. The data was fitted using linear regression and t_o/λ_a^o was obtained from the slopes. Thus, when λ_a^o is known, the overlayer thickness can be determined. Here, λ_a^o was calculated using the procedure developed for organic materials by Cumpson.[7]

A method for quantitative determination of adsorbed amount by means of XPS was initially developed for muscovite mica surfaces, where the known amount of exchangeable surface ions was used as reference.[8-10] The method was later extended to other substrates, such as cellulose,[8] gold,[11] and silica.[6] In our analysis, we instead utilize the known bulk chemical composition of calcite as reference, following the procedure described earlier and used for studying adsorption to silica surfaces.[6] Using this approach, the number of C1-carbon atoms from the stearic acid n_d (atoms/nm^3) on the calcite surface was calculated from **Eq. S3**:

$$n_d = \frac{I_d S_a n_a \, e^{\left(-\frac{t_o}{\lambda_a^o \sin\theta}\right)}}{I_a S_d \left[1 - e^{\left(-\frac{t_o}{\lambda_d^o \sin\theta}\right)}\right]} \quad \textbf{(S3)}$$

where I_d is the photoelectron peak intensity from element *d* in the overlayer (the C1-carbon contribution to the C 1s peak), S_d the relative sensitivity factor for element *d*, $\lambda_{d=C\,1s}^o$ the IMFP in the stearic acid overlayer for photoelectrons emitted from element *d* in the overlayer (calculated according to Cumpson).[7]

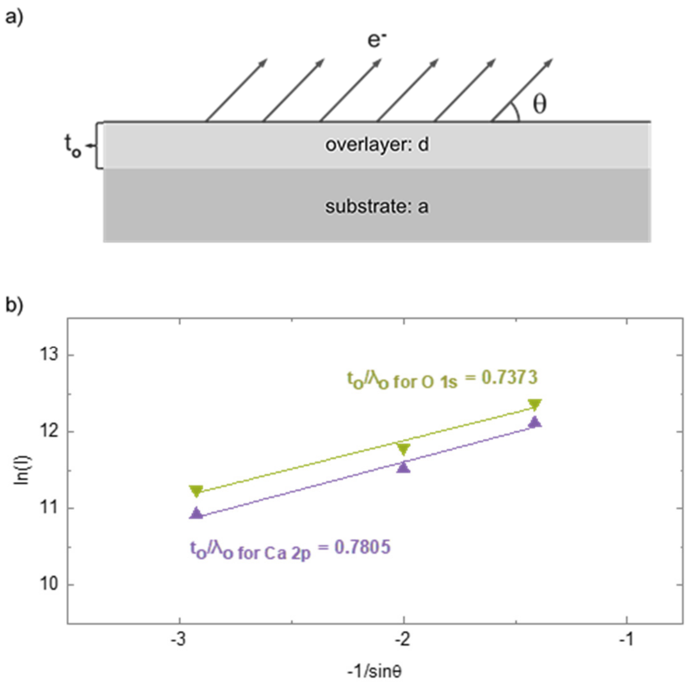

Figure S5. (a) Schematic diagram of the substate-overlayer model with photoelectrons e^- at take-off angle θ from the sample surface while assuming a homogeneous and fully covering stearic acid overlayer *d* with thickness t_o on a homogeneous-calcite substrate surface a. **(b)** The dependence of the natural logarithm of the peak intensity for the substrate signal (*I*) as a function of the photoelectron take-off angle (θ), where θ was 45°, 30 ° and 20°. For a calcite sample exposed to stearic acid vapour at 105 °C for 4 h, the data are shown for two substrate signals: Ca 2p (in violet), and for comparison O 1s (in olive). The linear fitting was used for calculating t_o/λ_a^o values).

The volume atomic density of the substrate element, n_a, was calculated from the structural formula of calcite, $CaCO_3$, with molecular weight 100.09 g/mol, Avogadro's number and the density of calcite (2.71 g/cm^3).[3] The calculated theoretical atomic densities for calcite were 16.3 Ca/nm^3, 16.3 C/nm^3 and 48.9 O/nm^3. In calcite, the theoretical atomic ratio O/Ca = 3, and after adjusting for oxygen present in the organic C2-C4-carbon peaks, the experimentally determined O/Ca atomic ratios were found to be close to the theoretical ratio. Therefore, we used the theoretical atomic density of 16.3 Ca/nm^3 in the calculations of adsorbed amount according to **Eq. S3**.

For quantification of the adsorbed amount of stearic acid expressed as stearic acid molecules/nm^2 the number of carbon atoms n_d (atoms/nm^3) was divided by 17, the number of C1-carbon atoms in one stearic acid molecule, and then multiplied with the layer thickness determined using **Eq. S2**. In the calculations, we considered two assumptions: i) none of the C1-carbon (aliphatic carbon) found on freshly cleaved calcite was displaced by stearic acid, and ii) all C1-carbon found on the freshly cleaved calcite surface was displaced by stearic acid.

XPS: Adsorption to calcite in ultra-high vacuum XPS conditions

We investigated the chemical stability of the freshly cleaved calcite surface by keeping it for almost 24 h in ultra-high vacuum conditions (pressure below $1.33 \cdot 10^{-5}$ Pa). The organic carbon on the surface increased by about 3.0 atomic % after 3 h under X-ray irradiation and a further increase by 1.5 atomic% after 24 h in high vacuum with X-ray irradiation. We can thus conclude that even under high vacuum, organic molecules physisorbed to the calcite surface, but the adsorption increase is small and occurs at a low rate.

XPS: Effects of X-ray irradiation

The possible damaging effect of X-ray irradiation on the stearic acid layer during long exposure times was evaluated (**Figure S6**) using calcite surfaces exposed to stearic acid vapour for 4 h. Here, we considered changes in the atomic ratio of the C 1s (total carbon) to Ca 2p elemental signals. The C 1s / Ca 2p atomic ratio decreased with X-ray exposure time, see **Figure S6**. This suggests fragmentation and/or desorption of the stearic acid layer either directly by X-ray radiation, by interactions with photoelectrons or due to heating of the sample surface and sample holder, which both could induce desorption. Heating of the sample holder has been noted in other studies after long X-ray irradiation times.[12]

As a result of continuous X-ray irradiation, the signal from the stearic acid layer decreased, whereas that from the calcite substrate increased. We note, however, that all XPS measurements for quantification of layer thickness and adsorbed amount were done after short X-ray irradiation exposure times (below 10 minutes, where the carbon to calcium ratio decreased just to about 95 % of the starting value, that is, the atomic ratio of $C_{(total\ carbon)}$ / Ca decreased from 4.21 at 5 min to 4.01 at 10 min). Any underestimation of thickness and adsorbed amount due to degradation is, therefore small.

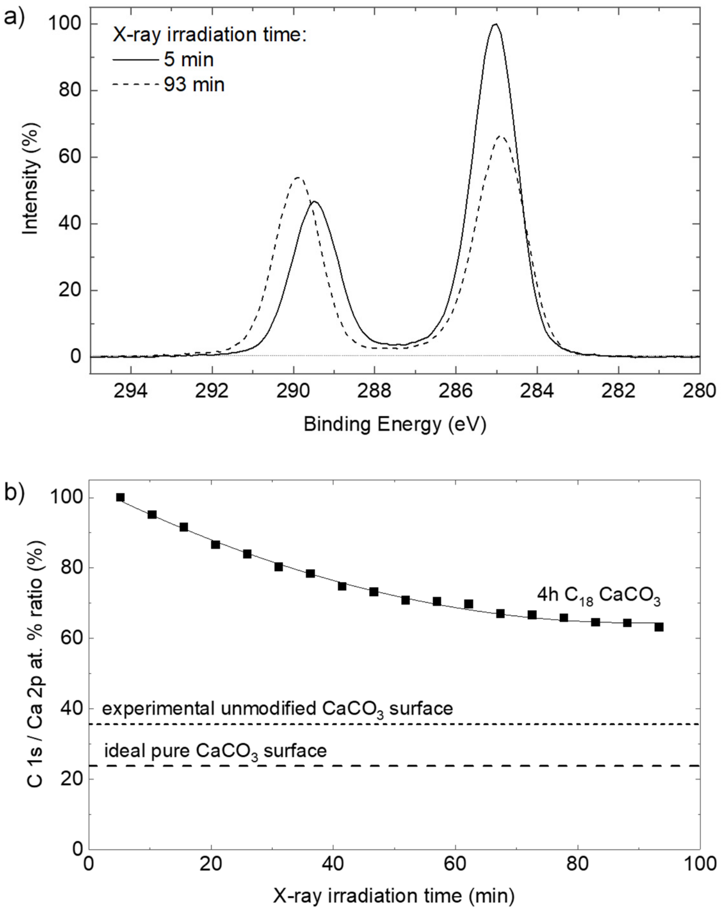

Figure S6. Degradation and / or desorption of the stearic acid layer in the XPS instrument under the influence of X-ray radiation. **(a)** The C 1s spectra evaluated during the first scan (straight line), and during the 18th scan 93 min later (dashed line). The sample was exposed to stearic acid vapor for 4 h at 105 °C. **(b)** The C 1s/ Ca 2p atomic ratio relative to the value at 5 min (4.21) as a function of X-ray irradiation time fitted with a polynomial function.

4. Vibrational sum frequency spectroscopy.

Sum frequency spectra of stearic acid adsorbed on the calcite surface after a 24 h exposure for all azimuthal angles measured under the SSP polarization are presented in **Figure S7**.

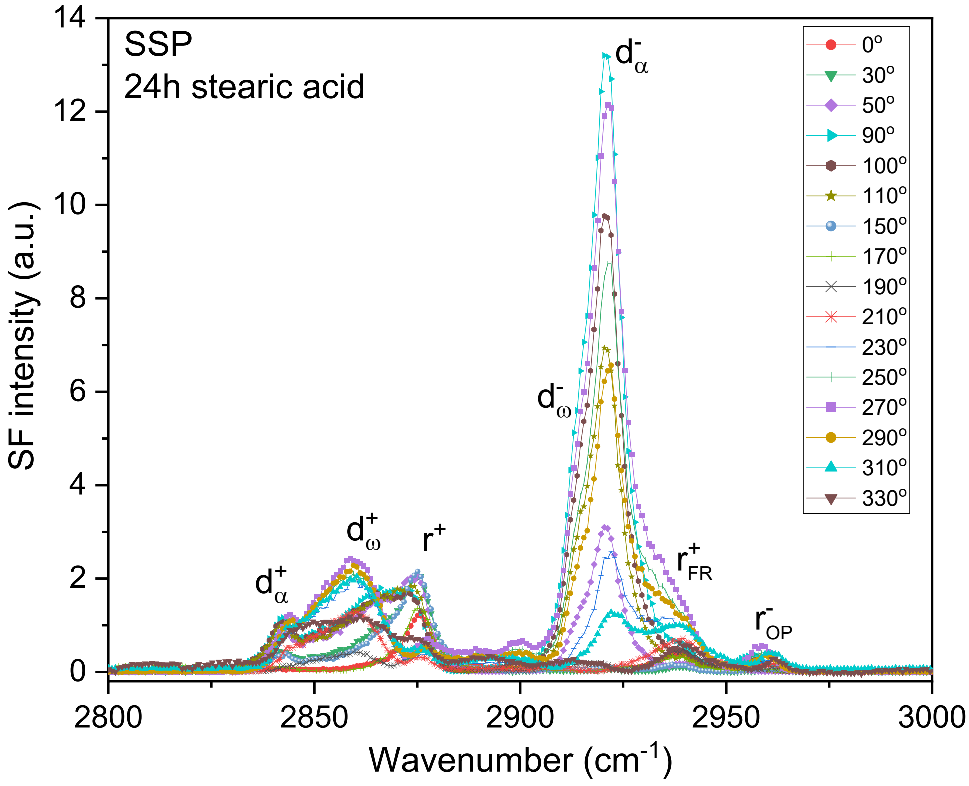

Figure S7. Vibrational sum frequency spectra of a stearic acid monolayer adsorbed (24 h) on calcite, measured as a function of the azimuthal angle (Ψ) as defined in the main article. Polarization combination: SSP.

The spectra were subsequently fitted using a convolution of Lorentzian and Gaussian line shapes of the form presented in **Eq. S4**. The Lorentzian and Gaussian components account for the homogeneous and inhomogeneous line broadening, respectively.[13]

$$I_{\mathrm{SF}} \propto |\, A_{\mathrm{NR}}^{(2)} + \sum_{v} \int_{-\infty}^{\infty} \frac{-A_v e^{\frac{(\omega_v'-\omega_v)^2}{2\sigma_v^2}}}{\sqrt{2\pi\sigma_v^2}(\omega_{\mathrm{IR}}-\omega_v'+i\Gamma_v)} d\omega_v' \,|^2 \quad \textbf{(S4)}$$

where A_{NR} represents the non-resonant contribution, A_v is the amplitude of vth resonant mode, ω_{IR} is the IR frequency and ω_v, Γ_v and σ_v are the peak position, Lorentzian width and Gaussian line width, respectively. The obtained fitting parameters for the most relevant bands at the different azimuthal angles are listed in **Table S4**. Note that the Lorentzian line widths were constrained to 2 cm^{-1}, while σ_v were allowed to vary between 1.8 – 3 cm^{-1}.

Table S4. Fitted amplitudes

Azimuthal angle Ψ in degrees	Fitted Amplitudes (arb. units) d_α^+ (2842±2 cm^{-1})	d_ω^+ (2865±2 cm^{-1})	r^+ (2874±2 cm^{-1})	d_ω^- (2912±2 cm^{-1})	d_α^- (2921±2 cm^{-1})	r_{FR}^+ (2941±2 cm^{-1})
350	-0.107 ± 0.006	0.185 ± 0.005	0.284 ± 0.013	0.026 ± 0.009	-0.067 ± 0.007	-0.242 ± 0.005
0	-0.010 ± 0.005	0.117 ± 0.006	0.307 ± 0.001	-0.010 ± 0.010	-0.005 ± 0.005	-0.212 ± 0.005
10	-0.081 ± 0.005	0.053 ± 0.004	0.344 ± 0.002	-0.003 ± 0.010	0.032 ± 0.008	-0.144 ± 0.004
30	-0.210 ± 0.002	0.220 ± 0.002	0.436 ± 0.001	0.036 ± 0.008	0.030 ± 0.009	-0.077 ± 0.004
50	-0.309 ± 0.004	0.335 ± 0.005	0.408 ± 0.003	-0.300 ± 0.011	0.555 ± 0.002	0.040 ± 0.006
70	-0.367 ± 0.005	0.361 ± 0.007	0.366 ± 0.006	-0.564 ± 0.003	0.954 ± 0.001	-0.095 ± 0.014
90	-0.370 ± 0.005	0.372 ± 0.007	0.368 ± 0.006	-0.665 ± 0.003	1.119 ± 0.001	-0.152 ± 0.006
100	-0.368 ± 0.004	0.360 ± 0.005	0.373 ± 0.004	-0.527 ± 0.002	0.937 ± 0.001	-0.113 ± 0.008
110	-0.326 ± 0.005	0.317 ± 0.006	0.421 ± 0.004	-0.376 ± 0.004	0.801 ± 0.001	-0.075 ± 0.011
130	-0.269 ± 0.004	0.306 ± 0.004	0.373 ± 0.003	-0.206 ± 0.004	0.294 ± 0.002	-0.083 ± 0.014
150	-0.153 ± 0.002	0.219 ± 0.003	0.420 ± 0.002	0.039 ± 0.006	0.012 ± 0.008	-0.095 ± 0.006
170	-0.120 ± 0.008	0.108 ± 0.008	0.326 ± 0.004	-0.004 ± 0.017	0.048 ± 0.016	-0.178 ± 0.007
190	-0.154 ± 0.003	0.230 ± 0.002	0.195 ± 0.002	0.049 ± 0.004	-0.041 ± 0.003	-0.230 ± 0.002
210	-0.290 ± 0.003	0.425 ± 0.002	0.131 ± 0.005	-0.036 ± 0.011	0.086 ± 0.014	-0.295 ± 0.003
230	-0.365 ± 0.005	0.521 ± 0.004	0.166 ± 0.007	0.095 ± 0.012	0.469 ± 0.008	-0.246 ± 0.008
250	-0.360 ± 0.008	0.545 ± 0.005	0.236 ± 0.009	-0.446 ± 0.004	0.958 ± 0.002	-0.319 ± 0.011
270	-0.383 ± 0.007	0.592 ± 0.005	0.244 ± 0.009	-0.515 ± 0.003	1.102 ± 0.002	-0.471 ± 0.006
290	-0.383 ± 0.006	0.553 ± 0.004	0.237 ± 0.008	-0.358 ± 0.003	0.789 ± 0.002	-0.321 ± 0.008
310	-0.366 ± 0.008	0.540 ± 0.007	0.207 ± 0.011	0.057 ± 0.026	0.247 ± 0.024	-0.247 ± 0.014
330	-0.253 ± 0.001	0.390 ± 0.006	0.235 ± 0.008	0.036 ± 0.016	-0.049 ± 0.015	-0.242 ± 0.010
350	-0.104 ± 0.003	0.183 ± 0.003	0.199 ± 0.002	0.082 ± 0.005	-0.031 ± 0.004	-0.182 ± 0.003

Sum frequency spectra collected at polarization combinations different to those shown in the main article are presented in **Figure S8**. Note, in particular, the SPP spectrum in **Figure S8b**. In an isotropic planar surface, the intensity in the latter polarization is expected to be zero.[14] However, the fact that peaks are detected provides additional proof that the stearic acid monolayer on calcite is anisotropic in the plane.

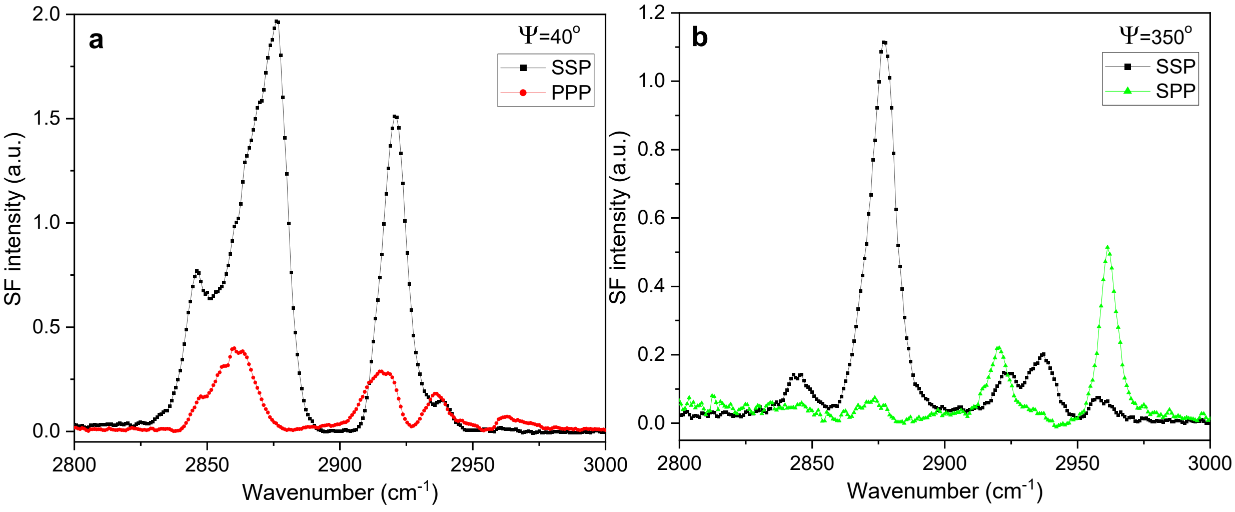

Figure S8. Vibrational sum frequency spectra of a stearic acid monolayer adsorbed (24 h) on calcite, measured at different polarization combinations for two selected azimuthal angles (Ψ).

Vibrational Sum Frequency. Fitting the anisotropy patterns in the polar plots

To help visualize the azimuthal angle dependence for the different vibrational modes detected in the CH stretching region, the patterns presented in **Figure 8** of the main paper were fitted according to **Eq. S5.** The fitted values are shown in **Table S5**. From the patterns shown in the polar plots, complemented with equivalent figures obtained at different polarization combinations, a detailed molecular orientational analysis of the adsorbed stearic acid monolayer can be performed.[15-17] These will be presented in a separate study.

$$y = A(1 + d_1\sin\Psi + d_2\cos 2\Psi + d_3\sin 3\Psi + d_4\cos 4\Psi) \quad \textbf{(S5)}$$

Table S5

	d_α^+ **(2842±2 cm^{-1})**	d_ω^+ **(2865±2 cm^{-1})**	r^+ **(2874±2 cm^{-1})**	d_ω^- **(2912±2 cm^{-1})**	d_α^- **(2921±2 cm^{-1})**
A	-0.27 ± 0.01	0.35 ± 0.01	0.30 ± 0.01	-0.16 ± 0.02	0.40 ± 0.02
d_1	0	-0.37 ± 0.03	0.38 ± 0.03	0.45 ± 0.16	0
d_2	-0.52 ± 0.05	-0.47 ± 0.02	0	-1.74 ± 0.22	-1.40 ± 0.09
d_3	0	-0.09 ± 0.03	0.19 ± 0.03	0	0
d_4	-0.16 ± 0.04	-0.17 ± 0.02	0	0.70 ± 0.17	0.34 ± 0.07

Vibrational Sum Frequency: intrinsic calcite modes

Figure S9 shows the sum frequency spectrum of a freshly cleaved calcite surface in the absence of stearic acid. The sharp feature observed in the spectra at 1432 cm^{-1} unambiguously stems from the calcite surface and is assigned to the antisymmetric carbonate stretch ($\nu_{3\ \mathrm{calcite}}$).[18]

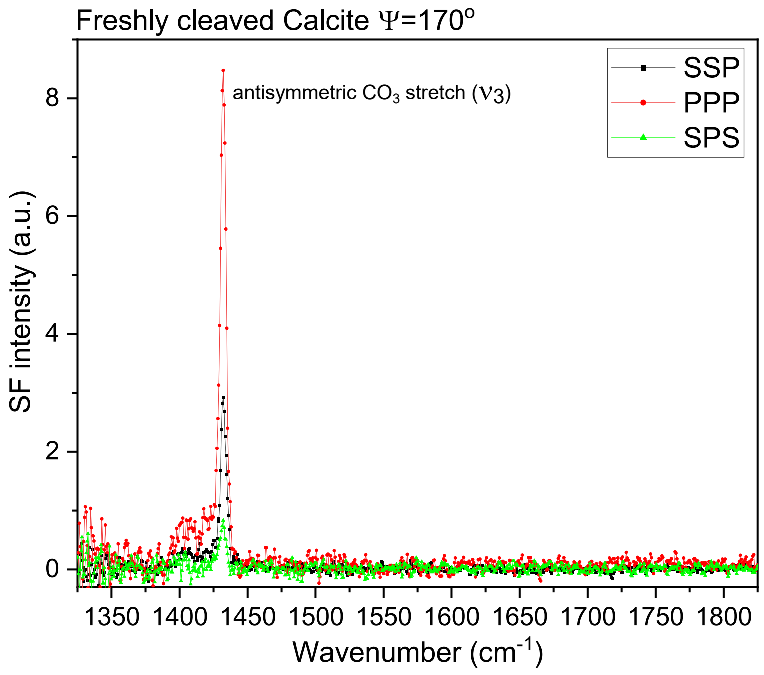

Figure S9. Vibrational sum frequency spectra of freshly cleaved and unmodified calcite sample at three different polarizations (without the stearic acid monolayer)

REFERENCES

1. Wilson, J. A.; Chickos, J. S., Vapor Pressures and Vaporization, Sublimation, and Fusion Enthalpies of Some Fatty Acids. *Journal of Chemical & Engineering Data* **2013,** *58* (2), 322-333.
2. de Kruif, C. G.; Schaake, R. C. F.; van Miltenburg, J. C.; van der Klauw, K.; Blok, J. G., Thermodynamic properties of the normal alkanoic acids III. Enthalpies of vaporization and vapour pressures of 13 normal alkanoic acids. *The Journal of Chemical Thermodynamics* **1982,** *14* (8), 791-798.
3. Rumble, J. R., CRC Handbook of Chemistry and Physics, 100th Edition (Internet Version 2019). Taylor and Francis: Boca Raton, FL: 2019.
4. Stull, D. R., Vapor pressure of pure substances. Organic and inorganic compounds. *Industrial & Engineering Chemistry* **1947,** *39* (4), 517-540.
5. Beamson, G.; Briggs, D., High Resolution XPS of Organic Polymers: The Scienta ESCA300 Database John Wiley & Sons. *Inc., New York* **1992**.
6. Ernstsson, M.; Claesson, P. M.; Shao, S. Y., Characterization of adsorption sites on a quartz powder from ESCA analysis of an adsorbed fatty diamine. *Surface and Interface Analysis: An International Journal devoted to the development and application of techniques for the analysis of surfaces, interfaces and thin films* **1999,** *27* (10), 915-929.
7. Cumpson, P. J., Estimation of inelastic mean free paths for polymers and other organic materials: use of quantitative structure–property relationships. *Surface and Interface Analysis: An International Journal devoted to the development and application of techniques for the analysis of surfaces, interfaces and thin films* **2001,** *31* (1), 23-34.
8. Rojas, O. J.; Ernstsson, M.; Neuman, R. D.; Claesson, P. M., X-ray photoelectron spectroscopy in the study of polyelectrolyte adsorption on mica and cellulose. *The Journal of Physical Chemistry B* **2000,** *104* (43), 10032-10042.
9. Claesson, P.; Herder, P.; Stenius, P.; Eriksson, J.; Pashley, R., An ESCA and AES study of ion-exchange on the basal plane of mica. *Journal of colloid and interface science* **1986,** *109* (1), 31-39.
10. Herder, P. C.; Claesson, P. M.; Herder, C. E., Adsorption of cationic surfactants on muscovite mica as quantified by means of ESCA. *Journal of colloid and interface science* **1987,** *119* (1), 155-167.
11. Plunkett, M. A.; Claesson, P. M.; Ernstsson, M.; Rutland, M. W., Comparison of the adsorption of different charge density polyelectrolytes: a quartz crystal microbalance and X-ray photoelectron spectroscopy study. *Langmuir* **2003,** *19* (11), 4673-4681.
12. Medvedev, N.; Tkachenko, V.; Lipp, V.; Li, Z.; Ziaja, B., Various damage mechanisms in carbon and silicon materials under femtosecond X-ray irradiation. *arXiv preprint arXiv:1805.07524* **2018**.
13. Bain, C. D.; Davies, P. B.; Ong, T. H.; Ward, R. N.; Brown, M. A., Quantitative analysis of monolayer composition by sum-frequency vibrational spectroscopy. *Langmuir* **1991,** *7* (8), 1563-6.
14. Beattie, D. A.; Fraenkel, R.; Winget, S. A.; Petersen, A.; Bain, C. D., Sum-Frequency Spectroscopy of a Monolayer of Zinc Arachidate at the Solid-Solid Interface. *J. Phys. Chem. B* **2006,** *110* (5), 2278-2292.
15. Nihonyanagi, S.; Miyamoto, D.; Idojiri, S.; Uosaki, K., Evidence for Epitaxial Arrangement and High Conformational Order of an Organic Monolayer on Si(111) by Sum Frequency Generation Spectroscopy. *J. Am. Chem. Soc.* **2004,** *126* (22), 7034-7040.
16. Malyk, S.; Shalhout, F. Y.; O'Leary, L. E.; Lewis, N. S.; Benderskii, A. V., Vibrational Sum Frequency Spectroscopic Investigation of the Azimuthal Anisotropy and Rotational Dynamics of Methyl-Terminated Silicon(111) Surfaces. *J. Phys. Chem. C* **2013,** *117* (2), 935-944.
17. Ge, A.; Rudshteyn, B.; Psciuk, B. T.; Xiao, D.; Song, J.; Anfuso, C. L.; Ricks, A. M.; Batista, V. S.; Lian, T., Surface-Induced Anisotropic Binding of a Rhenium CO2-Reduction Catalyst on Rutile TiO2(110) Surfaces. *J. Phys. Chem. C* **2016,** *120* (37), 20970-20977.
18. Bischoff, W. D.; Sharma, S. K.; MacKenzie, F. T., Carbonate ion disorder in synthetic and biogenic magnesian calcites: a Raman spectral study. *Am. Mineral.* **1985,** *70* (5-6), 581-589.
